# Mapping the nonribosomal specificity code through promiscuity-guided A-domain engineering

**DOI:** 10.1039/d6sc00250a

**Published:** 2026-02-26

**Authors:** Aleksa Stanišić, Carl-Magnus Svensson, Maximilian Müll, Freddy A. Bernal, Hannah Zeihe, Ulrich Ettelt, Hajo Kries

**Affiliations:** a Biosynthetic Design of Natural Products, Leibniz Institute for Natural Product Research and Infection Biology – Hans-Knöll-Institute (HKI) Beutenbergstraße 11a 07745 Jena Germany; b Applied Systems Biology, Leibniz Institute for Natural Product Research and Infection Biology – Hans-Knöll-Institute (HKI) Beutenbergstraße 11a 07745 Jena Germany; c Transfer Group Anti-Infectives, Leibniz Institute for Natural Product Research and Infection Biology – Hans-Knöll-Institute (HKI) Beutenbergstraße 11a 07745 Jena Germany; d Department of Technical Biochemistry, University of Stuttgart Allmandring 31 70569 Stuttgart Germany hajo.kries@ibc.uni-stuttgart.de

## Abstract

Nonribosomal peptide synthetases (NRPSs) assemble bioactive peptides from various building blocks. The binding pocket residues governing building block specificity have allowed prediction of NRPS products but not design of specificity. A reason for this failure has been ignorance of NRPS multispecificity. Here, we employ a hydroxamate assay (HAMA) to determine multispecificity for mutant libraries of the adenylation (A-)domain in module SrfAC of surfactin synthetase. A multispecific version of SrfAC is developed and its functional flexibility probed by fully randomizing 15 residues around the active site. We identify mutations with profound impact on specificity revealing remarkable evolvability and explain the effect of a selected mutant by computational modelling. Statistical analysis of the specificity divergence caused by 285 point mutations has revealed an outstanding influence of three sequence positions on specificity, which provides a roadmap for NRPS engineering. Our results suggest that promiscuity drives neofunctionalization of A-domains and mimicking this process will help to design valuable peptides in the lab.

## Abstract 2

From specialist to generalist and back: probing active-site mutations defines a roadmap for redesigning substrate specificity of adenylation domains from nonribsomal peptide synthesis.

## Graphical abstract

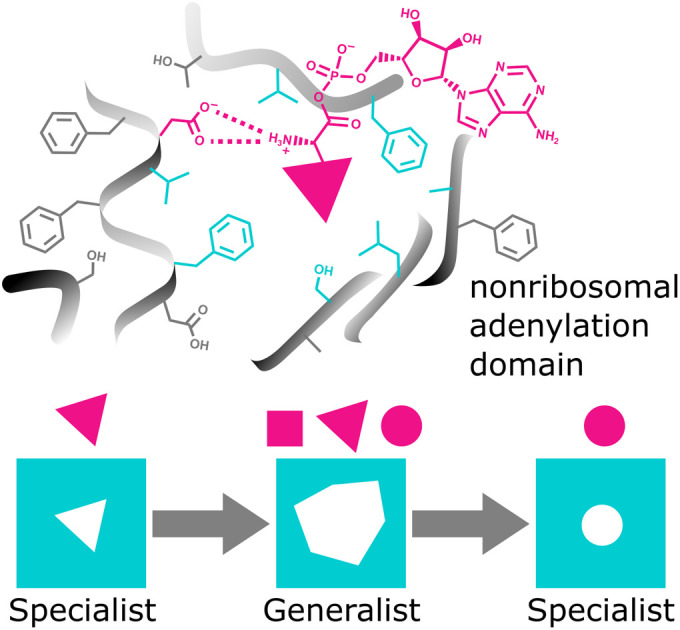

## Introduction

Promiscuous enzyme activities serve as evolutionary springboard towards novel functions.^1–3^ It is believed that in natural evolution, multispecific, generalist ancestor enzymes have gained specificity under strong selective pressure. Directed evolution imitates this process in the laboratory to design customized enzymes with broad applications.^4–9^ To generate suitable starting points for directed evolution, specialized enzymes can be reverted to promiscuous, ancestor-like states by amplifying weak activities towards noncognate substrates.^10^ These generalist enzymes then undergo re-specialization towards a desired function in laboratory evolution.^11^ Not all enzymes are equally evolvable, however. It has been shown that enzyme families with high natural functional diversity are more amenable to change than those fulfilling identical roles across the homology tree.^12^ Secondary metabolism is especially enriched with promiscuous activities resulting in a mishmash of natural product congeners.^13–15^ Hence, enzymes from secondary metabolism seem especially suitable for studying promiscuity and mapping evolutionary pathways between different functions.^16^

**Fig. 1:**
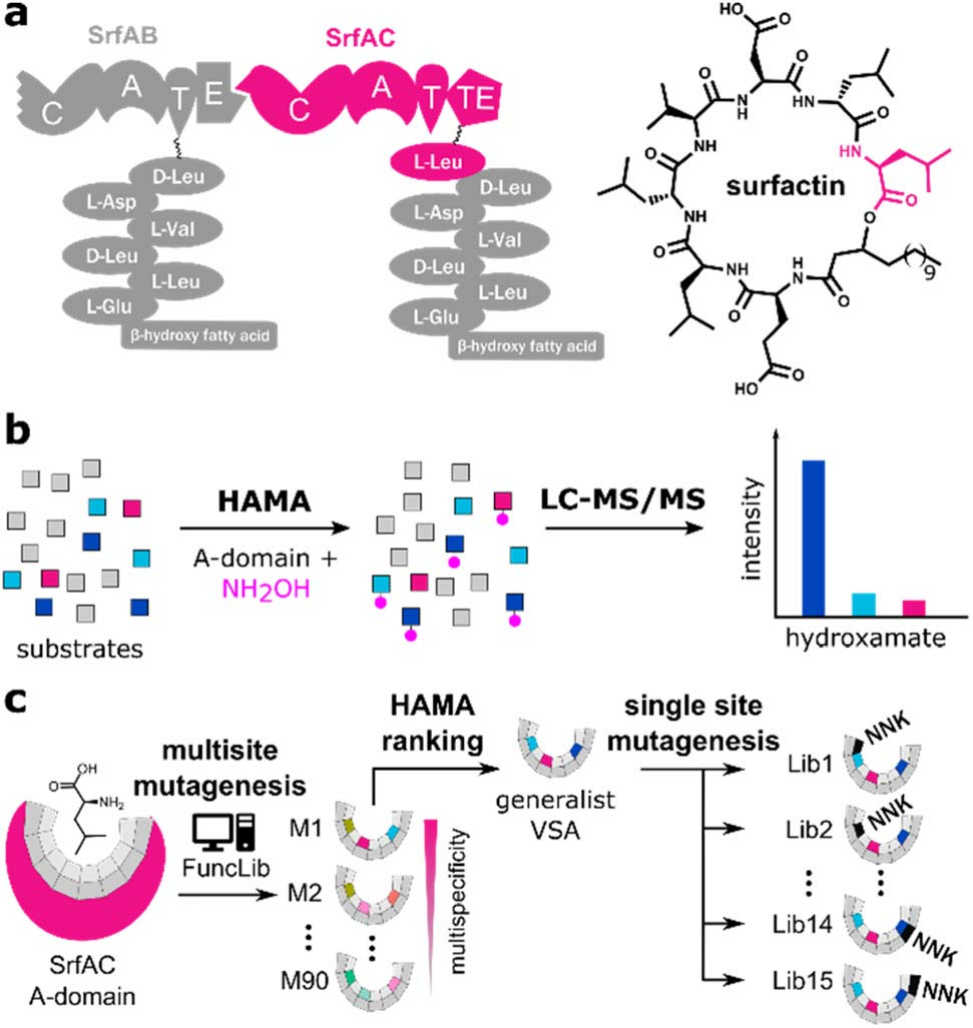
(a) The nonribosomal peptide synthetase module SrfAC incorporates the terminal Leu into surfactin. (b) The hydroxamate assay (HAMA) records substrate profiles of nonribosomal adenylation (A-)domains.^28^ (c) The substrate profile of l-Leu-specific SrfAC is diversified by multisite mutagenesis aided by the FuncLib algorithm (M1: mutant 1),^10^ followed by site-directed saturation mutagenesis at 15 positions close to the A-domain active site (Lib1: library 1) using degenerate NNK codons.

Nonribosomal peptides (NRPs) are a class of secondary metabolites of great importance for human use as antibiotics, immunosuppressants, and anti-cancer drugs.^17^ NRPs are assembled on large multidomain enzymes termed nonribosomal peptide synthetases (NRPSs).^18^ NRPSs consist of enzyme domains catalysing individual reactions. Domains are grouped in modules, each of which incorporates a single substrate into the peptide chain in an assembly line fashion (Fig. 1a). Substrates are first activated with ATP by adenylation (A-)domains before being tethered to thiolation (T-)domains and condensed with the substrate from the adjacent module by condensation (C-)domains. The release of the final product is typically achieved by a terminal thioesterase (TE-)domain catalysing hydrolysis or intramolecular cyclization of mature linear peptides. The large variety of NRPS architectures and corresponding NRP products must result from fast evolutionary diversification. Sequences of NRPS gene clusters suggest evolutionary mechanisms relying on a combination of gene recombination and neofunctionalization.^19–25^ However, the neofunctionalization mechanisms of individual modules have remained largely elusive because nonribosomal multispecificity has been cumbersome to analyze.^26,27^

The modular nature of NRPSs makes them an attractive engineering target for sourcing custom-made peptides. Controlling A-domains, which in turn control the identity of activated and incorporated substrates, would unlock efficient biosynthetic drug design.^29^ Natural A-domains recognize many more substrates than the proteinogenic amino acids^30–32^ and can be highly specific,^33^ bispecific,^34^ or multispecific.^16,21,35^ Structures and sequences have revealed the conservation of ‘specificity code’ residues in the binding pockets of A-domains activating the same substrate.^36–38^ The initial 8-residue code, later amended by 2nd and 3rd shell residues, allowed the development of algorithms predicting the identity of the final products from NRPS protein sequence.^32,39–41^

When NRPS reprogramming was attempted based on A-domain specificity codes, successful switches were limited to structurally similar substrates indicating that specificity signatures are not readily transferable between A-domains. Nevertheless, good designability of A-domain specificity has been demonstrated,^42,43^ for instance on Phe-specific GrsA (gramicidin S NRPS) which acquired a 5 × 10^5^-fold switch in specificity towards “click” amino acid propargyl-Tyr by introducing a single mutation in the binding pocket.^27,44^ Accompanying C-domains can sometimes prevent A-domain engineering from producing new peptide structures.^45^ However, growing evidence from structural biology,^46^ engineering,^47,48^ and natural evolution^19^ suggests that in many cases, A-domain engineering by itself will be sufficient. High-throughput screening using yeast surface display has further bypassed the limitations of rational A-domain design,^49,50^ but this approach remains limited to substrates with a covalent binding handle.

For efficient A-domain engineering, it is essential to better understand how binding pocket mutagenesis impacts specificity profiles. In previous work, we have developed a hydroxamate assay (HAMA, Fig. 1b) to determine a complete specificity profile of an A-domain in a single reaction, dramatically reducing the workload and facilitating the determination of A-domain multispecificity.^28^ In HAMA, hydroxylamine is added as a quencher to an adenylation reaction, where a set of substrates is presented to the A-domain. Stable hydroxamates are formed from the activated aminoacyl-AMP intermediates and those can be detected using UPLC-MS/MS. In contrast to conventional adenylation assays, HAMA reports on multiple substrates simultaneously under direct competition, mimicking intracellular conditions. Therefore, HAMA provides a more realistic picture of A-domain multispecificity than other assays and the hydroxamate profile has been demonstrated to be a good proxy not only for *k*_cat_/*K*_M_ profiles^28^ but also for predicting the peptide-formation specificity of NRPS mutants.^45,51^ Notably, the presence of 150 mM hydroxylamine in HAMA ensures that hydrolysis of the aminoacyl AMP intermediate^52^ does not determine the turnover rate. Possibly, the attack of hydroxylamine on the aminoacyl-AMP intermediate resembles the transacylation half-reaction so that a reaction is probed with high mechanistic relevance.

Here, we take advantage of HAMA to investigate the impact of mutations on the specificity landscape of the A-domain from SrfAC, the termination module from surfactin synthetase in *B. subtilis*. SrfAC is a standalone module with C-A-T-TE architecture incorporating the terminal l-Leu into the biosurfactant surfactin (Fig. 1a). We harness HAMA to unravel the evolutionary pathways leading towards diverse specificities *via* a generalist intermediate with broadened substrate spectrum (Fig. 1c). Computationally supported introduction of multisite mutations into module SrfAC has yielded a variant (VSA) with enhanced activity towards several substrates and retained stability. In a library of single mutations prepared from VSA, we demonstrate high flexibility of adenylation specificity. Quantitative understanding of the mutational landscape of NRPS specificity and a refined specificity code will serve as a roadmap for the future engineering of novel bioactive molecules.

## Results and discussion

### Making SrfAC multispecific

**Fig. 2:**
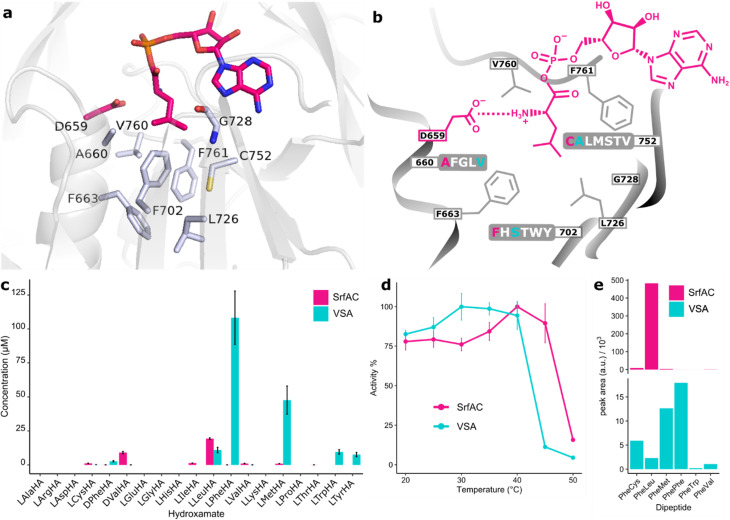
SrfAC and mutant VSA with relaxed specificity. (a) Binding pocket of SrfAC homology modelled with YASARA^57^ in complex with l-Leu-AMP (sticks in pink). Specificity code residues are shown as grey sticks. The model is built against the SrfAC crystal structure as a template (PDB: 2VSQ).^53^ (b) Mutagenesis of the SrfAC binding pocket. Three specificity code residues of SrfAC selected for randomization (660, 752, and 702) and the list of tolerated residues predicted by FuncLib^10^ are marked. SrfAC and VSA residues are labelled in pink and cyan, respectively. (c) Hydroxamate concentrations from HAMA specificity profiles. Enzyme reactions were incubated for 60 min at 25 °C with 1 µM enzyme and a mixture of 19 amino acids. (d) Thermostability enzyme reactions were incubated for 20 min at different temperatures and 1 µM enzyme and the production of hydroxamates was followed. Error bars are standard deviations from three (c) or two (d) technical replicates from two batches of enzyme. (e) SrfAC or VSA (0.5 µM) were combined with GrsA (1 µM) and incubated with a mixture of 19 amino acids for 2 h. Peptides were detected by triple quad LC-MS/MS using a Phe-derived fragment (*m*/*z* = 120) of the (M + H)^+^ parent ion. All detectable products are shown.

Assuming that promiscuous activities are evolutionary steppingstones towards novel functions, our first aim was to develop a variant of SrfAC with relaxed specificity (Fig. 2). Module SrfAC has been thoroughly investigated with biophysical methods,^53,54^ and shows stable expression in *E. coli* in microtiter plate format (Fig. S1). Interestingly, we found that SrfAC has substantial activity towards nonproteinogenic d-Val, which should be inconsequential to the host *B. subtilis* due to the absence of this stereoisomer in the organism. SrfAC shows further side activities *ca.* 20-fold (l-Ile, l-Cys, l-Val, l-Met) or 340-fold (l-Phe) lower than the main activity for l-Leu (Fig. 2c). In nature, surfactins are produced as a mixture of congeners, including [Val_7_]surfactin,^55,56^ which presumably results from the weak side-activity of the SrfAC A-domain. Good protein behaviour combined with weak natural multispecificity make this system ideal for studying NRPS promiscuity.

Multisite mutations can functionally diversify enzyme active sites but have a high risk to be deleterious for activity and stability. To gain a functional repertoire of diverse SrfAC multisite mutants, we took advantage of FuncLib.^10^ This automated algorithm uses phylogenetic analysis and Rosetta modelling to predict the tolerability of mutations. FuncLib filters out mutations likely to result in inactive variants. To do so, FuncLib scores single site mutants present in a multiple sequence alignment using the Rosetta algorithm, which offers a good approximation of protein stability.^58^ As input for the FuncLib webserver, SrfAC in complex with l-Leu-AMP was modelled using the YASARA software (Fig. S2).^59^ The l-Leu-AMP ligand and the invariable catalytic residue D659 (Fig. 2a) were fixed during FuncLib calculations. From the eight positions of the specificity code (A660, F663, F702, L726, G728, C752, V760, F761), we selected three for experimental multisite randomization based on experience from previous A-domain engineering campaigns.^27,49^ Being located at the entrance (A660 and C752) and the bottom (F702) of the binding pocket, a decisive impact on substrate recognition was expected. At these three positions, the most beneficial 5 (A660), 6 (F702), and 7 (C752) residue identities according to the first stage of the FuncLib protocol were combined in a random library (Fig. 2b). The library of 210 triple mutants was cloned by combining oligonucleotides bearing degenerate codons for each position in appropriate ratios (Tables S1 and S2) and using them for overlap-extension PCR. As expected for an A-domain activating a nonpolar amino acid, FuncLib predicted mutational tolerance towards residues with predominantly aliphatic or hydrophobic side chains (Fig. 2b).

To analyze the triple mutants, protein was expressed in four 96-well microtiter plates from randomly picked colonies and specificity profiles with 17 proteinogenic and two nonproteinogenic substrates were measured with HAMA. The strength of the FuncLib prediction is illustrated by 46% of library members having detectable activity – remarkably high considering significant losses in activity typically accompanying multisite A-domain mutagenesis. Out of the candidates with highest activity and promiscuity, triple mutant A660**V**-F702**S**-C752**A** (VSA) was selected for further characterization (Table S6). VSA shows enhanced activity for several substrates and activates l-Phe and l-Met in HAMA at rates surpassing SrfAC with l-Leu. Additionally, activity of VSA for l-Leu, l-Trp, and l-Tyr is high (Fig. 2c). The large-to-small mutation F702S presumably frees up space for bulkier, hydrophobic amino acid substrates such as l-Phe and l-Met. The enhanced turnover for multiple substrates predisposes VSA for further diversification because the high activity lifts several substrates above the detection threshold.

In addition to broad substrate tolerance, an ancestor-like enzyme must be stable enough to withstand further mutations.^60^ To test thermal stability, the adenylation activity of VSA was followed at a range of temperatures between 20 and 50 °C (Fig. 2d). VSA maintains full activity up to 40 °C, indicating only minor stability trade-offs in comparison with the parent SrfAC. To characterize the effect of the VSA mutations, saturation kinetics with the three major substrates (l-Leu, l-Phe and l-Met) were measured with the MesG/hydroxylamine assay (Fig. S3). The adenylation rate *k*_cat_ for all three substrates is maintained at wild type-like levels with differences originating in *K*_M_ values. *K*_M_(l-Leu) shows a 50-fold increase from 0.013 ± 0.001 mM in SrfAC to 0.510 ± 0.050 mM in VSA, while the *K*_M_'s of VSA for l-Phe and l-Met (1.3 ± 0.1 mM, 4.6 ± 0.9 mM, respectively) are within a 10-fold range of l-Leu. Consequently, specificity constants *k*_cat_/*K*_M_ of all three VSA-substrates fall within one order of magnitude. In combination with Phe-specific NRPS module GrsA as a starter module, the competence of VSA to form peptides with broadened specificity was confirmed and again, the major substrates were l-Met and l-Phe (Fig. 2e), but at a *ca.* 30-fold lower peptide production level. The increased hydroxamate signal observed for VSA in HAMA, contrasting with the reduced peptide formation, likely reflects that HAMA reports adenylation only. Productive peptide synthesis also depends on downstream steps that can become kinetic bottlenecks with alternative substrates and thereby reduce final product yield.

Following the paradigm that generalist enzymes are hubs of natural evolution,^1,3,11,61^ we have de-engineered the NRPS module SrfAC into multispecific VSA as a stepping stone towards more diverse specificities. Compared to SrfAC, the triple mutant VSA shows remarkable increases for l-Met and l-Phe, and a generally higher level of hydroxamate formation with promiscuous substrates (Fig. 2c). Combining good stability and an expanded substrate repertoire at wild type-like adenylation activity, VSA was well suited for further functional diversification.

### Making GrsA multispecific

To test whether other NRPS modules can be made multispecific as easily as SrfAC, we followed an analogous mutational strategy with the well-studied model NRPS GrsA. GrsA is the first module from the gramicidin S synthetase and from its A-domain structure^36^ the NRPS specificity code has been deduced. The positions corresponding to the VSA mutations were identified as 236, 278, and 322. For these positions, suitable mutations were again predicted using FuncLib, mutations were combinatorially combined using PCR primers with degenerate codon sets, and protein variants were screened in 96-well plate format. In this fashion, variant GYG was identified carrying mutations A236G, T278Y, and A322G. Here, in contrast to the SrfAC variant VSA, the overall hydroxamate formation suffered a 13-fold reduction, but an expansion of the substrate profile was again detected and confirmed with protein purified on a larger scale (Fig. S4). In GYG, Trp became the best substrate along with His and the wild-type substrate Phe.

### Broad functional sequence space in VSA mutants

**Fig. 3:**
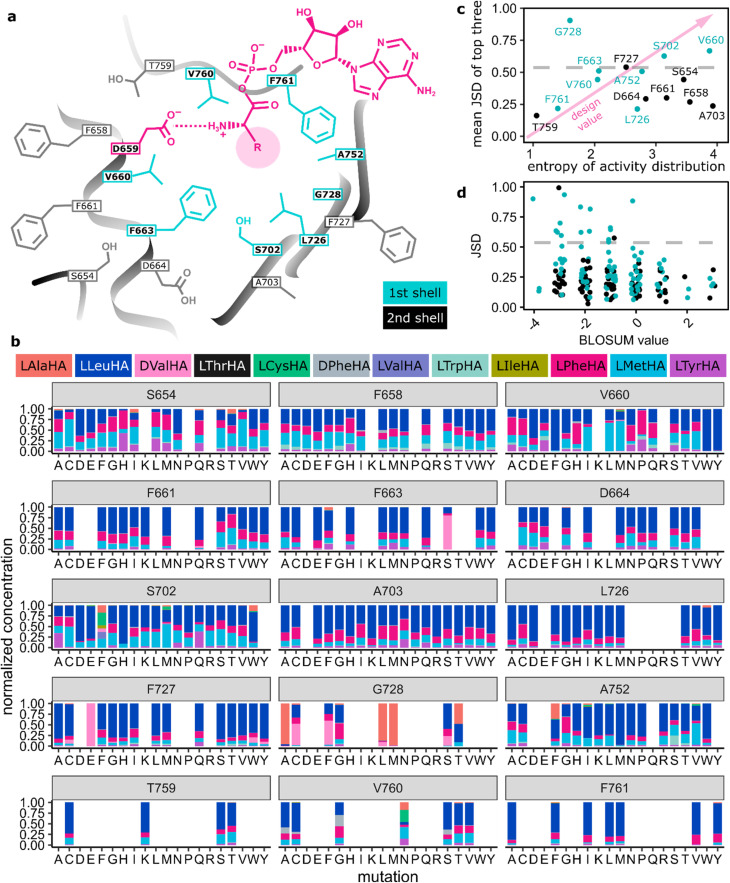
(a) Scheme of the VSA binding pocket. “First shell” is synonymous with the specificity code defined in ref. 37 and 38. (b) HAMA specificity profiles from screening NNK libraries of VSA. When the sum of hydroxamates for one mutant is below 1% of the wild type, it is set to zero; sums of hydroxamates for all other mutants are normalized to one. Identical mutants have been averaged. (c) Average Jensen–Shannon distance (JSD) of the top three mutants per position^62^ (S654: LHD, F658: LQH, V660: WYF, F661: QTS, F663: QSE, D664: MPT, S702: HWF, A703: KNT, L726: WCV, F727: CYE, G728: ALM, A752: IMF, T759: SCK, V760: GAN, F761: AVY) plotted against the entropy of the activity distribution at the same position (cyan: first shell, black: second shell). The dashed line indicates significant divergence from VSA. (d) JSD for each mutant plotted against the BLOSUM score of the mutation, where more negative values indicate a larger mutational distance.

Having established VSA as a robust mutant with broad specificity, we proceeded to thoroughly probe the effects of single point mutations on the specificity profile of VSA. We exhaustively covered binding pocket and surroundings with site-saturation mutagenesis libraries in 15 positions (Fig. 3a, S5 and Tables S3, S4). In addition to the 8 specificity code residues in the first shell, 7 second shell residues were included. To maintain 90% coverage of each NNK library, we screened 92 colonies per library with HAMA in microtiter plates. Libraries were sequenced and completed by individually cloning mutants missing from them (Table S5). Activity levels were higher than 1% of the wild-type in 66% (187/285) of mutants from all libraries. Out of 19 offered substrates, 11 yielded detectable product with at least some of the mutants (Fig. 3b and S6). The set of active substrates was strongly biased towards low polarity but, interestingly, included d-configured Phe and Val (Fig. S6).

### Ranking positions for designability

By recording the specificity profiles of the A-domain mutants, we wanted to learn in which positions mutations achieved the largest specificity changes. Therefore, we ranked all 15 positions according to the largest difference in specificity profile obtained with mutants at that position (Fig. 3c). The difference in specificity was quantified as the Jensen–Shannon distance (JSD, SI)^62^ of the hydroxamate distributions. The JSD was chosen as a metric because it is symmetric with respect to the direction of the comparison and bounded to the interval 0 to 1. Mutants with less than 1% of the wild type activity were excluded from the analysis to avoid artefacts. To identify the most “designable” positions, the three mutants for each position with the largest JSD were averaged. Only three positions (660, 702, 728) out of the 8 canonical code positions in the first shell of the binding pocket showed significant deviations from VSA in the top-three-average. A notable omission is position 663, which showed an overwhelming impact on specificity in the GrsA and TycA modules,^27^ but is just below the significance threshold in the ranking here. Mutations in the second shell did not change specificity profiles significantly. Therefore, by analysing even more distant residues, we would not expect fundamentally new insights for the designability ranking, even if finely tuned long-range interactions can be important to design a perfect catalyst.^63^

We expect the three prioritized positions to be most useful for reprogramming A-domain specificity in structurally similar proteins and for chemically similar amino acid substrates. For example, the transition from α- to β-Phe specificity, required loop remodelling^49^ and would not be likely to be achieved by mutating the three priority positions, which are all located in the rigid secondary structure elements surrounding the binding pocket. However, if these secondary structures are conserved, as is the case even for fungal A-domains,^64^ there is a good chance that the design principles discovered here may still apply, but this hypothesis requires experimental validation. Notably, also partner domains (C-, E- or TE-domain) can constrain substrate acceptance and co-engineering may be necessary to arrive at the final natural product analogue.

In addition to the divergence of specificity, the activity level of mutants is an important parameter to gauge the usefulness of a position for A-domain design (Fig. S7 and S8). Activity distributions for all mutations in one position were represented as entropy values (Fig. 3c). A high entropy value indicates a uniform distribution of activity across mutations and therefore high mutational tolerance. Low entropy values indicate sensitive positions where all mutations strongly reduce activity compared to the wild type. The most useful positions for design will combine a large divergence of the specificity profile with robust activity (top right corner of Fig. 3c). For instance, first shell positions 702 in the β-sheet and 660 adjacent to the conserved D659 have been identified as particularly valuable for design, because they give rise to diverse specificity at good activity levels. At the other extreme, at position T759, activity is almost destroyed already by a conservative mutation to Ser without yielding a noticeable difference in specificity. As expected, specificity profiles generally diverged more strongly from the starting point when mutations were less conservative, as measured by the BLOSUM score (Fig. 3d). Accordingly, polar (Asp, Glu, His, Lys, Arg), rigid (Pro), and bulky (Trp, Tyr) substitutions in the hydrophobic A-domain binding pocket create mostly inactive enzymes (Fig. S7).

### Hit validation

**Fig. 4:**
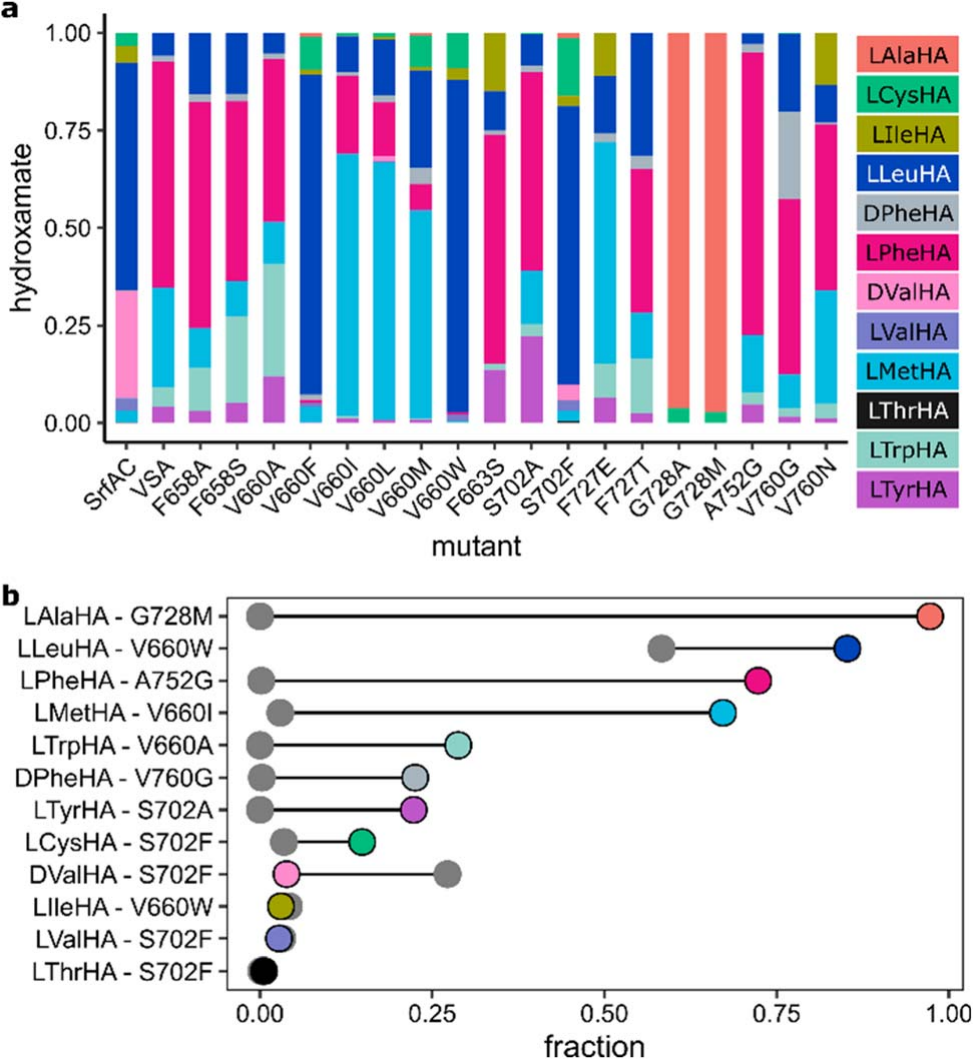
Validation of screening hits. (a) Normalized HAMA specificity profiles of selected VSA mutants from large scale purification. Enzyme reactions were incubated for 60 min at 25 °C and 1 µM purified enzyme. (b) For each hydroxamate, the mutant showing the largest fraction in its product spectrum (colored circle) is compared to SrfAC (grey). For unnormalized activity levels, see Fig. S9.

Due to the large technical variability in our multistep microtiter plate screening protocol, we remeasured HAMA profiles for selected mutants after large-scale purification (Fig. 4) to challenge the most remarkable results. Thus, it was confirmed that two mutants at position G728 (G728A, G728M) show high specificity for Ala, which is not a detectable substrate in SrfAC or VSA. Another mutant (V660W) achieves higher specificity for Leu than SrfAC without side-activity for d-Val. l-Met is rarely encountered as NRPS building block but has been enhanced already in VSA compared to SrfAC. Several mutations in position 660 make l-Met the major substrate (*e.g.* V660I). Additionally, activation of d-configured Phe is favoured by Gly substitution at V760. The strongly enhanced promiscuity of the S702F mutant and the full switch to d-Val in F727E observed in the screening experiment could not be corroborated in the validation experiment (Fig. S10 and 4a). Curiously, neither l-Val nor l-Ile showed improved activity with any mutant despite their similarity to l-Leu. Apparently, the β-branched structure of these amino acids constitutes a larger difference for molecular recognition than the overall chemical similarity to l-Leu suggests. The course away from β-branching may have been set early in the experiment by choosing VSA as a multispecific intermediate, which already stops recognizing d-Val.

Although half of single mutants showed activity with at least one aliphatic amino acid, no hydroxamates of polar amino acids were detected. This suggests that multiple binding pocket residues must co-adapt to accommodate different physicochemical substrate classes. Consequently, transitioning to a new substrate class may require combinatorial mutagenesis – possibly together with co-engineering of partner C-, E- or TE-domains and targeted product screening – to identify synergistic mutation combinations that bypass such fitness valleys. Within one class of substrate, specificity switches are remarkably easy to achieve. With reference to SrfAC, full specificity switches (l-Ala, l-Phe, l-Met) or substantial improvements (l-Trp, d-Phe, l-Tyr, l-Cys) were confirmed for seven substrates (Fig. 4b). Notably, our screening method has uncovered binding pockets that do not exist in nature (Table S7 and Fig. S16), for instance one for the substrate l-Leu with Trp in position 660 (Fig. 4). Natural specificity codes for l-Leu have Ala in position 660 in 94% of the cases but never Trp. The potential for discovering novel codes for the same substrate has earlier been demonstrated by Throckmorton *et al.*^65^

### Molecular simulation of the G728M mutational effect

**Fig. 5:**
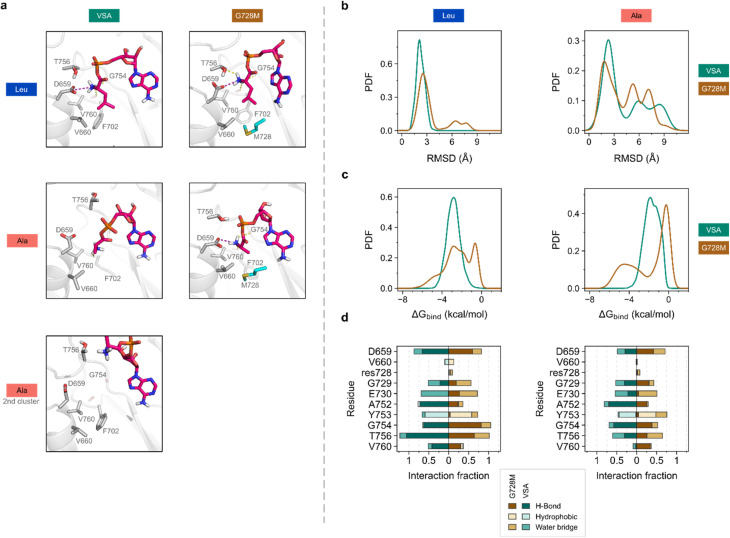
Structural analysis of VSA and mutant G728M by unbiased MD simulations. (a) Representative binding modes of l-Leu-AMP or l-Ala-AMP derived from MD trajectory clustering (top and middle) and another representative cluster for the VSA – l-Ala-AMP complex (bottom). Substrate shown in pink liquorice. Representative binding site residues in grey. For simplicity, only key interactions are shown (yellow for H-bond; purple for ionic interaction). (b) Distribution of substrate RMSD values along MD simulations. (c) Distribution of the contribution of residue 728 to the calculated binding free energy. Distributions in c and d are expressed as probability density functions (PDFs). (d) Substrate–protein interactions with representative residues along MD simulations res728 = residue 728 (Gly728 or Met728 in VSA and mutant, respectively). Clustering and PDF values calculated on four MD replicates of 200 ns (see Fig. S11 and S13). Binding free energy calculated by MM/GBSA method.

The high specificity of mutant G728M for l-Ala, which is not present in SrfAC or VSA, prompted us to analyze the ligand–protein complex at the molecular level. We used unbiased molecular dynamics (MD) simulations to explore both conformational space and key interactions for l-Leu-AMP and l-Ala-AMP ligands binding to VSA and G728M (Fig. 5). As expected, both ligands showed differential intermolecular interactions upon binding. Clustering of the MD trajectories based on substrate RMSD demonstrated that the four studied systems were able to establish strong interactions – *i.e.*, the binding mode appeared rather conserved for the largest cluster in each case (Fig. 5a). However, additional conformational motifs were identified for VSA – l-Ala-AMP, whereby important polar contacts were significantly modified, resulting in a highly solvent exposed pose (Fig. 5a, bottom). In general, l-Ala-AMP as ligand underwent significant conformational changes over the simulation as demonstrated by a large range of RMSD values, indicating high instability, compared to l-Leu-AMP-containing systems (Fig. 5b and S11). End-point binding free energy calculations along the MD trajectories could not explain the observed differences between systems (Fig. S12). However, residue decomposition analysis indeed suggested that the mutated residue 728 was significantly affecting the calculated binding energy. Met728 contributed to the overall binding energy with l-Ala-AMP more strongly than Gly728 at least for a subset of substrate conformations (Fig. 5c and S13).

A fundamental interaction in A-domains binding α-amino acids is that between the α-amino group and the carboxylate of the conserved residue Asp659. Indeed, mutation G728M caused an increased number of interactions between the l-Ala-AMP ligand and D659 along the simulations (Fig. 5d). Additionally, in G728M, l-Ala-AMP was able to maintain interactions with Val760, which were almost totally depleted in VSA (Fig. 5d and S14). Backbone hydrogen bonding by Val760 located in a loop at the back of the active site apparently complements amino group binding by Asp659. The importance of Val760 for substrate binding agrees with mounting evidence that the loop carrying this residue Val760 is an important hot-spot for A-domain engineering, when the substrate structure is modified at the α-amino group.^44,49^ Another backbone amide in the same loop, Gly754, located higher in the binding pocket, also interacts with the substrate amino group but does not show the same potential for discrimination (Fig. 5d and S15).

The hydrogen bond between the substrate amino group and the backbone carbonyl of residue 760 (Fig. 5) is a previously underappreciated interaction that may play a key role in substrate binding. This hydrogen bond, formed with an almost immovable backbone carbonyl, appears to act as a checkpoint: it is only established when the substrate side chain is fully inserted into the binding pocket. Interestingly, in the VSA variant, the weakly bound l-Ala-AMP ligand can slide out of the pocket without significantly disrupting the frequently discussed interaction with conserved Asp659, suggesting that this backbone hydrogen bond at position 760 may be a critical determinant of tight substrate binding.

## Conclusion

In summary, these insights will help to prioritise positions for the directed evolution of A-domain specificity in the future. We demonstrate the strength of promiscuity-guided screening to create generalist A-domains as progenitors for further diversification. Introducing point mutations in multispecific VSA at only a few positions has been sufficient to achieve large changes in specificity, often without impairment in activity. The specificity and activity profiles for 15 positions in the A-domain binding pocket region have allowed to prioritize three highly designable positions, which will serve as a roadmap for redesigning nonribosomal peptides. Considering the ease by which single mutations switch the specificity of A-domains, we predict that screening smart, medium-sized libraries focusing on those priority positions will suffice to unlock various target substrates.^66,67^ This remarkable plasticity of A-domain specificity also explains how evolution has been able to recruit a smorgasbord of building blocks for nonribosomal peptides.

## Materials and methods

### Protein models

The 3D-model of SrfAC in complex with an l-Leu-AMP ligand was built with YASARA.^68^ The model was aligned to the crystal structure of SrfAC (PDB: 2VSQ)^53^ to confirm that modelling did not change the position of specificity code residues in the binding pocket (Fig. S2). The 3D-model of the VSA mutant was created on the SWISS-MODEL server (https://swissmodel.expasy.org/)^69,70^ by using the crystal structure of SrfAC (PDB: 2VSQ) in its thiolation state as a template. Both models were then aligned in PyMOL (https://pymol.org/).

### Cloning

For the transformation of In-Fusion cloning reactions, *E. coli* HST08 Stellar competent cells were used (Takara Bio Europe). For the propagation and storage of plasmids, *E. coli* NEB 5-alpha (New England Biolabs) was used. Plasmid DNA, DNA fragments, and PCR products were purified using NucleoSpin kits (Macherey Nagel). DNA amplification was done with Q5 polymerase (New England Biolabs) following the supplier's instructions. Two-fragment cloning in linearized vector was done using the In-Fusion cloning kit (Takara Bio Europe). Oligonucleotide primers were made by custom synthesis (Eurofins Genomics) and sequence confirmation of assembled constructs was performed using the Mix2Seq service for Sanger sequencing. The plasmid pTrc99a-srfAC was kindly provided by Prof. Donald Hilvert (ETH Zurich). For the linearization of pTrc99a-srfAC, restriction enzymes BlpI, DraIII and BstBI (New England Biolabs, Massachusetts) were used, depending on the position of the mutation. Libraries were generated by amplification of one or two DNA fragments with primers bearing randomized codons. If the insert was amplified in two fragments, these were concatenated by assembly PCR to increase cloning efficiency. Cloned libraries were transformed into *E. coli* HST08 Stellar competent cells. Libraries were purified from overnight liquid cultures grown under ampicillin selection and resulting plasmid DNA was used to transform the protein expression strain *E. coli* HM0079.^71^ The identity of purified plasmids was confirmed by Sanger sequencing (Eurofins Genomics). The identity of library mutants was determined by Sanger sequencing of 96-well plates (Microsynth) containing aliquots taken from the saturated preculture shortly before induction of protein expression.

### FuncLib library of SrfAC

To identify the essential residues required for adenylation, a SWISS homology model of the SrfAC A-domain was built on the crystal structure of EntF (PDB: 5T3D) solved in complex with serine adenosine vinylsulfonamide inhibitor (Ser-AVS), a nonhydrolyzable analogue of serine-AMP. A structure of l-Leu-AMP was modelled into the SrfAC homology model using YASARA. The resulting model was used for FuncLib randomization.^10^ Informed by the structural model, eight specificity code residues of SrfAC were selected for simultaneous randomization by FuncLib: A660, F663, F702, L726, G728, C752, V760 and F761 at default parameters for multiple sequence alignments (min ID: 35, max targets: 4000, coverage: 75, *E* value: 0.0001). Conformations of AMP and D659 were fixed to maintain the interactions necessary for adenylation. FuncLib generated signatures of residues tolerated at each of the 8 selected positions. Three positions were selected (in bold) for the construction of a library of triple mutants (Table 1).

**Table 1:** Mutations of SrfAC prioritized by FuncLib

**A660: AFGLV**	F663: FIMWY	**F702: FHSTWY**	L726: LIMVY
G728: GACS	**C752: CALMSTV**	V760: VACFILTY	F761: FHILMWY

For generating a library of SrfAC triple mutants inspired by FuncLib, a series of oligonucleotides containing degenerate codons coding for predicted residues at the selected positions 660, 702, and 752 was used. The wild type residue was included in each position. For each randomized position, degenerate oligonucleotides were combined in ratios reflecting the number of possible codons and used for PCR amplification of DNA fragments with pTrc99a-SrfAC as a template (Table S1). A single DNA fragment for each position was generated resulting in three fragments (A, B, C). These were assembled by PCR using two primers with vector-specific overhangs (SrfAC_o_f and SrfAC_o_r, Table S2). Using In-Fusion, the assembled fragment was cloned into pTrc99a-SrfAC linearized with DraIII and BstBI, followed by transformation of Stellar competent cells. After the SOC outgrowth phase, 10 µL of the transformed culture was inoculated in 3 mL of TB medium containing ampicillin and grown overnight. The plasmid library purified from this culture was transformed into HM0079 for protein expression or NEB 5-alpha for long term storage.

### FuncLib library of GrsA

The process established on SrfAC was repeated on GrsA from the gramicidin S NRPS.^72^ The crystal structure of GrsA-A (PDB: 1AMU) was aligned with the SrfAC/Leu-AMP homology model. The l-Leu-AMP ligand was mutated to l-Phe-AMP and the model energy minimized with YASARA. The eight canonical specificity code residues of GrsA were selected for simultaneous randomization by FuncLib: A236, W239, T278, I299, A301, A322, I330 and I331. Conformations of Phe-AMP and D235 were fixed to maintain the interactions necessary for adenylation. FuncLib generated signatures of residues tolerated at each of the 8 selected positions. The same three positions randomized in SrfAC were selected (in bold) for construction of a library of triple mutants (Table 2).

**Table 2:** Mutations of GrsA prioritized by FuncLib

**A236: ALG**	W239: W	**T278: FVLTYIHASD**
I299: IVT	A301: AGST	**A322: ALCMVDTSEQNIGH**
I330: TFIM/NSV	I331: CVTAISNME	

The GrsA-236-278-322 library was constructed through a three-step cloning process. First, four individual fragments were amplified using specific primers, with linearized pSU18-mGrsA (0.5 ng µL^−1^) as the template.^27^ Following amplification, fragments were treated with DpnI, incubated for 1 h, heat-inactivated at 80 °C for 20 min, and purified *via* agarose gel electrophoresis. In the second step, the fragments were assembled using PCR with Q5 High-Fidelity DNA polymerase. A touchdown thermocycler program was used to accommodate varying annealing temperatures. For the final step, the assembly PCR product was fused with the PCR-amplified pSU18 vector *via* In-Fusion cloning and transformed into *E. coli* Stellar cells. Plasmid libraries were extracted and introduced into *E. coli* HM0079.

### NNK libraries of VSA

In the VSA gene, 15 positions coding for binding pocket residues were individually targeted for randomization with NNK codons to generate 15 libraries. DNA fragments containing NNK codons were amplified by PCR using pTrc99a-SrfAC-VSA as a template and NNK oligonucleotides as primers. During PCR with NNK oligos, amplification bias might have favoured codons similar to the wild type sequence and thus compromised library quality. To reduce amplification bias, silent mutations adjacent to the NNK codon were added. Depending on the location of the NNK codon in the gene, fragments were generated in one or two steps (Table S3). Where NNK positions were distant from both restriction sites, two fragments were generated and then assembled by PCR using two primers with vector-specific overhangs (VSA_Blp_f and SrfAC_o_r). In-Fusion cloning was done with one insert fragment carrying the NNK codon and one pTrc99a-SrfAC-VSA vector fragment linearized with appropriate restriction enzymes. Mutants missing from the libraries were cloned and screened in a separate sample batch (Table S5).

### Protein overexpression and purification

For the large-scale expression and purification of individual C-terminally His_6_-tagged holo-NRPS proteins, a saturated *E. coli* HM0079 (ref. 71) culture (0.5 mL) with the appropriate pTrc99a-SrfAC construct was inoculated in 500 mL of 2xYT medium supplemented with ampicillin in a 2 L shaking flask and shaken at 37 °C at 200 rpm. Cultures were grown for 4–6 h until OD_600_ = 1, induced with 0.25 mM isopropyl β-d-thiogalactopyranoside (IPTG) and grown for another 16–20 h at 20 °C. Cells were pelleted by centrifugation at 8000 g and the supernatant was discarded. Cell pellets were resuspended in 30 mL lysis buffer (50 mM Tris [pH 7.4], 500 mM NaCl, 20 mM imidazole, 2 mM tris(2-carboxyethyl)phosphine [TCEP]). Before cell lysis by sonication, 100 µL of protease inhibitor mix (Sigma, P8849) was added. The lysate was cleared by centrifugation at 19 000 g for 30 min at 4 °C. Proteins were applied to 2 mL of Ni-IDA suspension (Rotigarose, Roth) preequilibrated with lysis buffer by loading the lysate supernatant on the open column. Unbound proteins were removed by washing twice with 20 mL lysis buffer before protein was eluted with 4 × 0.75 mL elution buffer (50 mM tris [pH 7.4], 500 mM NaCl, 300 mM imidazole, 2 mM TCEP). Fractions containing protein were pooled and the buffer was exchanged with protein storage buffer (50 mM Tris [pH 7.6], 200 mM NaCl) on 6 mL Vivaspin (Sartorius) filters with 30 kDa cut-off. Glycerol was added to 10% and the protein concentration adjusted to 50 µM. Samples were flash frozen in liquid nitrogen and stored at −20 °C. To determine the protein concentration, absorbance at 280 nm was measured in Take3 plates on an Epoch2 microplate reader (Biotek) and converted into concentrations using calculated extinction coefficients (https://www.benchling.com/).

### HAMA

HAMA was conducted with purified proteins as described previously to determine multispecificity.^28^ Adenylation reactions of 100 µL contained 50 mM Tris (pH 7.6), 5 mM MgCl_2_, 150 mM hydroxylamine (pH 7.5–8, adjusted with NaOH), 5 mM ATP (A2383, Sigma), 1 mM TCEP and 1–5 µM of enzyme. Master mix without the enzyme was prepared and the reaction was initiated by adding enzyme or heat-inactivated enzyme as a control. l-Phe, l-Val and l-Leu were distinguished from d-Phe, d-Val and l-Ile, respectively, by using enantiopure, deuterium labelled standards (l-Phe-d5, l-Val-d8, l-Leu-d7; EQ Laboratories). As amino acid substrates, reactions generally contained l-Ala, l-Arg, l-Asp, l-Cys, l-Glu, l-Gly, l-His, l-Ile, l-Lys, l-Met, d-Phe, l-Pro, l-Thr, l-Trp, l-Tyr, d-Val, l-Val-d8, l-Phe-d5, and l-Leu-d7. For LC-MS/MS acquisition parameters, see ref. 28. Reactions were quenched after 1 h by 10-fold dilution in acetonitrile containing 0.1% formic acid and immediately analyzed by UPLC-MS/MS. All assays were done from two protein batches in technical triplicates unless otherwise indicated.

### UPLC-MS/MS detection of hydroxamates

Chromatography was performed on a Waters ACQUITY H-class UPLC system (Waters) after injecting 3 µL of the sample. Water with 0.1% formic acid (A) and acetonitrile with 0.1% formic acid (B) were used as strong and weak eluent, respectively. Separation of amino acid hydroxamates was achieved on a ACQUITY UPLC BEH Amide column (1.7 µm, 2.1 × 50 mm) with a linear gradient of 10–50% A over 5 min (flow rate 0.4 mL min^−1^) followed by 4 min re-equilibration. Data were analyzed with MassLynx and TargetLynx software (version 4.1). MS/MS detection was performed in positive ion mode on a Xevo TQ-S micro (Waters) tandem quadrupole instrument equipped with an ESI ionisation source. Nitrogen was used as desolvation gas and argon as collision gas. The following source parameters were used: capillary voltage 1.5 kV, cone voltage 65 V, desolvation temperature 500 °C, and desolvation gas flow 1000 L h^−1^. Specific mass transitions recorded in multiple reaction monitoring (MRM) mode were used to detect and quantify amino acid hydroxamates.^28^

### HAMA in 96-well plate format

*E. coli* HM0079 transformed with pTrc99a library constructs was used for overexpression of SrfAC variants in 96-well plate format. Precultures were prepared by inoculating the transformants picked from an agar plate into a round bottom 96-well plate (310 µL Sarstedt) filled with 150 µL of 2xYT medium supplemented with 100 µg mL^−1^ of ampicillin. Each 96-well plate contained four wells with a positive control (pTrc99a-SrfAC for FuncLib library, pTrc99a-SrfAC-VSA for NNK libraries) and 4 wells with a negative control (pTrc99a-SrfAC encoding for SrfAC with disrupted A-domain). Plates were covered with a breathable polyurethane film (Breathe-Easy, Sigma-Aldrich) and incubated for 18 h at 30 °C and 300 rpm in an orbital shaker. The following liquid handling steps were typically performed using a Gilson Platemaster 220 µL as 96-well pipette. For protein expression, 20 µL of the preculture was inoculated into a 96 deep-well plate (2 mL, Sarstedt) containing 1 mL 2xYT medium supplemented with 100 µg mL^−1^ ampicillin and incubated for 4–6 h at 30 °C and 300 rpm until the OD_600_ reached approximately 1. Prior to induction, a 20 µL aliquot was taken from the culture for preparing a 25% glycerol stock for long-term storage at −80 °C. Additionally, a 5 µL aliquot was taken for sequencing. For induction, the temperature was reduced to 18 °C for 30 min and 0.25 mM IPTG was added (Thermo Scientific). Incubation was continued at 18 °C and 300 rpm for 18–20 h. Cells were harvested by centrifugation at 3000 g and 15 °C and the supernatant was discarded. Immediately before lysis, 50 mL lysis buffer (50 mM Tris [pH 8.0], 100 mM NaCl, 10 mM imidazole, 1.5 mg mL^−1^ lysozyme) was prepared for each plate by adding 50 µL of protease inhibitor mix (P8849, Sigma). The pellet was resuspended in 400 µL lysis buffer per well and the plate was incubated for 30 min at room temperature. Cells were lysed by a single freeze-thaw cycle at −20 °C concluded by thawing the frozen plate for 1.5–2 h at room temperature.

After thawing, 100 µL of DNA removal mix (50 mM Tris [pH 8.0], 100 mM NaCl, 10 mM imidazole, 10 mM MgCl_2_, 10 mM TCEP, 15 U mL^−1^ Turbonuclease [Jena Bioscience]) was added to reduce the viscosity of the lysate and incubated without shaking at room temperature for 15 min. Cell debris was removed by centrifugation at 3000 g and 6 °C for 30 min. In a separate, 96-well plate (1.8 mL, Sarstedt) compatible with the magnetic separation rack (S1511S, New England Biolabs), 20 µL of a 25% Ni-IDA MagBeads (PureCube) suspension was added. The beads were equilibrated with 700 µL lysis buffer and the supernatant was discarded. To purify the released His_6_-tagged proteins from the lysate, 400 µL of the lysate supernatant was transferred to the equilibrated beads. The plate was covered with a silicon lid and kept at 4 °C in the fridge for 20 min with vigorous shaking every 5 min to prevent aggregation of the MagBeads. Beads were subsequently pulled down with a magnetic separator (S1511S, New England Biolabs) and the supernatant was discarded. To remove unbound protein and imidazole, the beads were washed twice with 700 µL of wash buffer (50 mM Tris [pH 8.0], 100 mM NaCl) with the help of the magnetic separator.

We found that enzymes maintain adenylation activity on beads so that elution was not necessary. The on-bead assay format was chosen because the imidazole from the elution buffer interferes with subsequent hydroxamate detection. After the second washing step, 100 µL of freshly prepared HAMA master mix (50 mM Tris [pH 8.0], 5 mM ATP, 5 mM MgCl_2_, 100 mM hydroxylamine [adjusted to pH 7.5–8 with NaOH], 1 mM TCEP, 1 mM of each substrate amino acid as above) was added directly to the beads containing the adsorbed protein and incubated at room temperature for 1.5 h. After incubation, 6 µL of the reaction mixture was diluted in 54 µL of analysis solution (95% acetonitrile, 0.1% formic acid, 1 µM pipecolic acid hydroxamate as an injection control) in a 384-well plate (100 µL, Brandt). After the dilution step, the 384-well plate was immediately placed on ice and covered with aluminum foil without glue to minimize evaporation of the solvent. The plate was analysed immediately by UPLC-MS/MS according to the general HAMA procedure.

### Thermostability assay

Thermal stability of SrfAC mutants from the FuncLib library was determined by measuring hydroxamate formation by HAMA after performing reactions for 20 min at different temperatures between 20 and 50 °C. Assays were done with two batches of enzyme in two technical replicates each.

### Dipeptide formation assay

GrsA was produced and purified as described previously.^51^ Reactions with 100 µL volume were prepared in a reaction buffer containing 50 mM HEPES (Roth), 50 mM MgCl_2_ (Roth), 1 mM TCEP (Iris Biotech), 5 mM ATP (Jena Biosciences), and 1 mM of a set of proteinogenic amino acids (Roth), along with 1 µM GrsA, at pH 7.5. Next, 0.5 µM of SrfAC, VSA or the respective heat-denatured control was added to the reaction. Denaturation was achieved by heating the enzyme at 70 °C for 10 min. The reactions were incubated at 25 °C for 2 h using a Veriti 96-well Fast Thermal Cycler (Applied Biosystems). Reactions were quenched by adding 100 µL of methanol, then cooled to 4 °C and centrifuged at 16 000 g for 20 min to remove the precipitate. The supernatant was diluted 1 : 5 in 50% ethanol/water containing 0.1% formic acid.

### UPLC-MS/MS detection of dipeptides

**Scheme 1:**
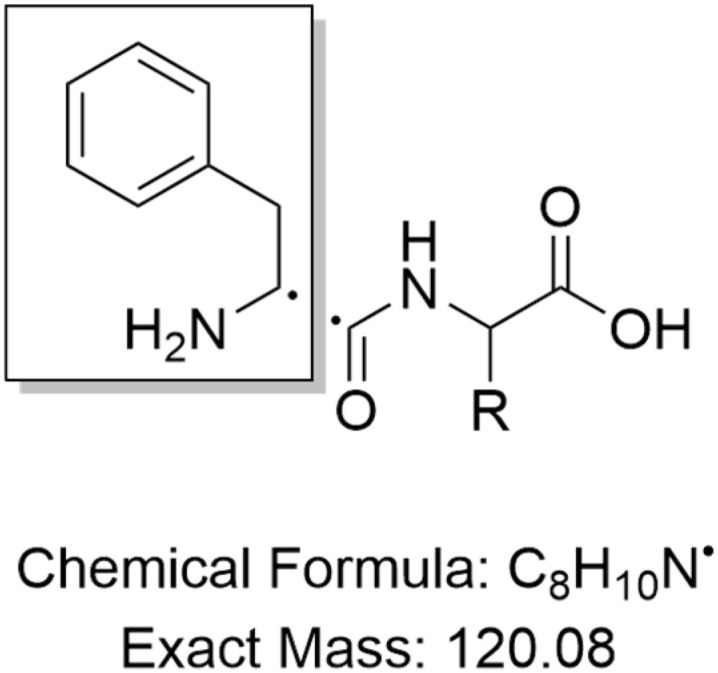
Fragmentation of Phe-Xaa dipeptides.

A 2 µL aliquot of each sample was injected into a UPLC H-Class system (Waters) using an FTN sample manager (Waters) and analyzed with a gradient of water + 0.1% formic acid (A) and acetonitrile + 0.1% formic acid (B), increasing from 2% B to 98% B over 3.2 min, followed by 1 min wash and re-equilibration. The column used was a 5 cm Cortecs C18 column (Waters), equipped with a VanGuard Cortecs C18 pre-column (Waters). The mass spectrometer used was a Xevo TQ-S micro (Waters) with a capillary voltage of 1.5 kV, a cone voltage of 65 V, a desolvation temperature of 500 °C, and a desolvation gas flow of 1000 L h^−1^. Nitrogen was used as the desolvation gas and argon as the collision gas. The experimental mode was set to multiple reaction monitoring (MRM), and the dipeptides Phe–Phe (*m*/*z* = 313.1), Phe–Trp (*m*/*z* = 352.1), Phe–Tyr (*m*/*z* = 329.1), Phe–Met (*m*/*z* = 297.1), Phe–Leu (*m*/*z* = 279.1), Phe–Ser (*m*/*z* = 269.1), and Phe–Val (*m*/*z* = 265.1) were all monitored with a transition to 120 Da, representing the common Phe-derived fragment shared by all analyzed peptides (Scheme 1). The collision energy for fragmentation was set to 24 V for all peptides. MRMs were run with a dwell time of 0.22 s.

### Saturation kinetics (MesG/hydroxylamine assay)

Michaelis–Menten parameters of the adenylation with l-Leu for SrfAC and additionally with l-Met and l-Phe for VSA were determined using the MesG/hydroxylamine assay.^73^ Low activity of SrfAC for l-Phe and l-Met prevented the determination of kinetic parameters. Reactions contained 50 mM Tris (pH 7.6), 5 mM MgCl_2_, 100 µM 7-methylthioguanosine (MesG), 150 mM hydroxylamine (adjusted to pH 7.5–8 with NaOH), 5 mM ATP (A2383, Sigma), 1 mM TCEP, 0.4 U mL^−1^ inorganic pyrophosphatase (I1643, Sigma), 1 U mL^−1^ of purine nucleoside phosphorylase from microorganisms (N8264, Sigma) and 5 µM of enzyme. Flat-bottom 384-well plates (100 µL, 781 620, Brand) were used for the reactions. Reactions were started by addition of enzyme and the absorbance was followed at 355 nm on a Synergy H1 (BioTek) microplate reader at 30 °C. Reactions used for background subtraction contained heat-inactivated enzyme. Each substrate concentration was measured in duplicate. Initial velocities (OD min^−1^) were divided by the slope of a pyrophosphate calibration curve to obtain the pyrophosphate release rate. Initial velocities *v*_0_/[*E*_0_] were fit to the Michaelis–Menten equation by nonlinear regression using *R*.^74^

### Statistical activity data analysis

Random sampling of colonies resulted in a variable number of replicates per mutant. Hydroxamate concentrations were averaged for these replicates within each batch of samples. The total activity of each mutant was calculated as a sum of *N*_H_ = 19 measured hydroxamates. To minimize the systematic error caused by variable protein expression and purification efficiency between different sample batches, the wild type control measured in each sample batch was used for normalization. A relative activity *A*_rel,m_ of each mutant m was calculated by dividing the total activity by the total activity of the wild type (eqn (1)).1
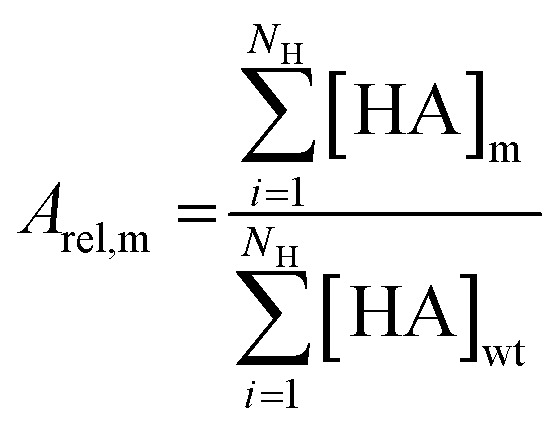


The promiscuity of each mutant was calculated based on the model proposed by Nath *et al.*^75^ The model uses the Shannon entropy *P* as a metric for promiscuity (eqn (2)) with *p*_*i*_ being the probability that the *i*'th substrate is converted to a hydroxamate by the enzyme.2
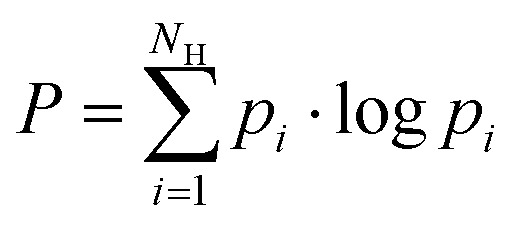


The probability *p*_*i*_ was derived from the proportion of the amino acid hydroxamates (eqn (3)).3
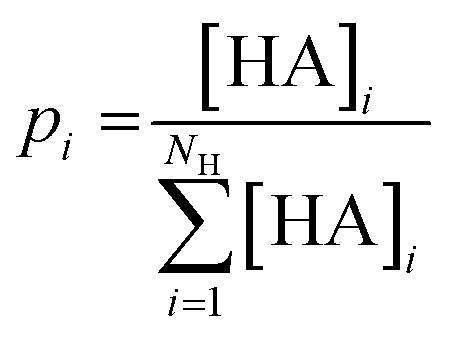


Based on *P*, the promiscuity index *I* of each mutant was calculated as follows:4
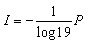


*N* indicates the number of measured hydroxamates (*N* = 19). The promiscuity index *I* can take values between 0 and 1, with 0 corresponding to perfectly specific and 1 to a perfectly promiscuous enzyme. To better discern the changes in promiscuity caused by mutations, a relative promiscuity index, *I*_rel_, was calculated by normalization to the wild type (eqn (5)). This results in values of *I*_rel_ larger than 1 for mutants more promiscuous, and lower than 1 for mutants more specific than the wild type.5
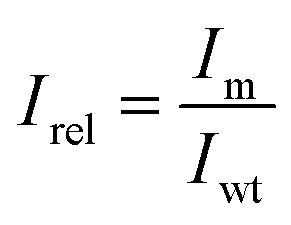


Due to the different detection limits of hydroxamates, low adenylation activity results in specificity profiles showing traces of individual products, which results in seemingly low *I*-values. To prevent the false designation of mutants as specific only because promiscuous activities are below the limit of detection, a cut off value for the activity was included. To filter out the mutants showing only traces of activity, before the promiscuity index was calculated, all mutants which accumulate less than 0.2 µM hydroxamates were excluded. Mutants were subsequently ranked according to *I*. *P* and *I* were calculated and visualized in *R* (Table S6).

The overall change in activity at one position has been measured as the entropy of the histograms for relative activities (eqn (1)) across all 20 mutations in one position. A low entropy value indicates that the overall activity changes a lot when the position is mutated, which usually results from most mutations reducing the activity and only the wild type remaining active.

To detect specific mutations that strongly change the substrate profile, we calculated the Jensen–Shannon distance (JSD) between the hydroxamates and the WT production which is bounded between 0 and 1.^62^ Given two probability distributions *P* and *Q*, we calculated the average distribution 
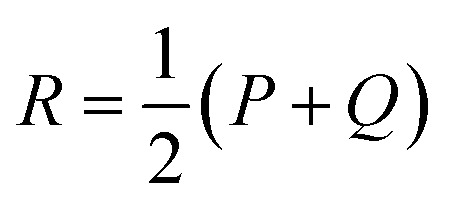
 and then6
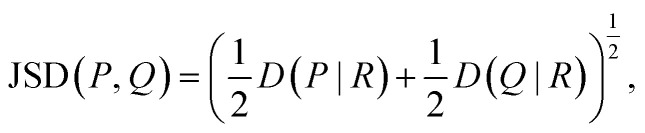
where *D*(*X*|*Y*) denotes the Kullback–Leibler distance between two distributions. The Kullback–Leibler distance was calculated as follows:7
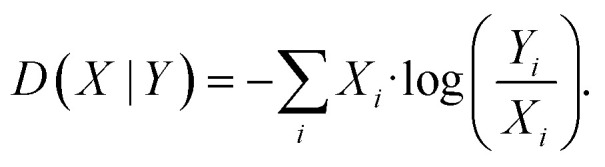


To avoid that low yielding hydroxamates cause artefacts, it was required that the total activity should be at least 1% of the WT production to be considered for JSD calculation. To determine whether the JSD for a mutation differs significantly from WT, the 95th percentile level of JSD between WT experiments was calculated for WT measured on the same 96-well plate.

### Molecular modeling

The model of SrfAC described earlier was prepared in Maestro (Schrödinger Suite, version 2022-2) using the Protein Preparation Workflow with default parameters. The H-bond network was refined by the sampling water orientation algorithm at pH of 7.4 using PROPKA.^76^ The structure was optimized by restrained energy minimization using the OPLS4 force field,^77^ with convergence criterion of 0.3 Å for heavy atoms RMSD. Manual residue mutation in Maestro conducted to the structures of VSA and its G728M mutant, which were subsequently energy minimized as described above.

MD simulations were performed using Desmond (Schrödinger release 2022-2) with the OPLS4 force field. The prepared structure for each complex was solvated using the simple point charge (SPC) water model in an orthorhombic simulation box with a 10 Å water buffer around the complex structure. The appropriate number of Na^+^/Cl^−^ counterions were added to neutralize the system and to reach a physiological salt concentration of 0.15 M. A default equilibration protocol was used prior production runs, including solute relaxation at *T* = 10 K using the NVT ensemble, solute relaxation at *T* = 10 K for 12 ps using the NPT ensemble, solute equilibration at *T* = 300 K for 12 ps, and NPT system equilibration without restraints at *T* = 300 K for 24 ps. Afterward, four production run replicates in the NPT ensemble at 300 K and 1 atm were carried out for 200 ns, changing the random seeds for the initial velocities. The Nosé–Hoover thermostat^78^ and the Martyna–Tobias–Klein barostat^79^ were used with default settings. A RESPA integrator was used with 2 fs timestep. Electrostatic forces were treated using the particle-mesh Ewald method^80^ with a default cut-off radius of 9 Å.

Protein–substrate interactions and conformational analysis were performed using the Simulation Interactions Diagram panel from Maestro and using in-house Python scripts based on the Schrödinger's Python API. MD triplicates were merged for each system and trajectory clustering was accomplished with the Desmond Trajectory Clustering tool in Maestro, using the affinity propagation clustering method,^81^ based on RMSD of the substrate atoms. Binding free energy was estimated for each trajectory frame with Prime^82,83^ using the molecular mechanics/generalized Born surface area (MM/GBSA) method^84,85^ as implemented in Maestro.

## Author contributions

H. K. and A. S. designed the project. A. S., M. M., H. Z., and U. E. performed experiments in the laboratory. C. M. S. carried out statistical analyses. F. A. B. performed molecular simulations. A. S. wrote the first draft. All authors contributed to data analysis, writing and revising the manuscript. H. K. supervised the project and secured funding.

## Conflicts of interest

There are no conflicts of interest to declare.

## Supplementary Material

SC-OLF-D6SC00250A-s001

## Data Availability

Datasets and code have been uploaded to https://github.com/applied-systems-biology/SrfAC. Supplementary information (SI): additional data analysis, tables, figures, and references. See DOI: https://doi.org/10.1039/d6sc00250a.
