# Supplementary material for: Mapping the nonribosomal specificity code through promiscuity-guided A-domain engineering
Source: Chem Sci. 2026 Feb 26;17(16):8168–81. doi: 10.1039/d6sc00250a (PMC12959268; doi:10.1039/d6sc00250a)
Supplement: SC-017-D6SC00250A-s001 [file SC-017-D6SC00250A-s001.pdf]

Supplementary Information (SI) for Chemical Science.
This journal is © The Royal Society of Chemistry 2026

Supporting Information

Mapping the Nonribosomal Specificity Code through Promiscuity-Guided A-Domain Engineering

Aleksa Stanišić,[a] Carl-Magnus Svensson,[b] Maximilian Müll,[a] Freddy A. Bernal,[c] Hannah Zeihe,[a] Ulrich Ettelt,[a] and Hajo Kries[a,d*]

[a] Dr. A. Stanišić, M. Müll, H. Zeihe, U. Ettelt, Dr. H. Kries
Biosynthetic Design of Natural Products,
Leibniz Institute for Natural Product Research and Infection Biology – Hans-Knöll-Institute (HKI),
Beutenbergstraße 11a, 07745 Jena, Germany

[b] Dr. C.-M. Svensson
Applied Systems Biology,
Leibniz Institute for Natural Product Research and Infection Biology – Hans-Knöll-Institute (HKI),
Beutenbergstraße 11a, 07745 Jena, Germany

[c] Dr. F. A. Bernal
Transfer Group Anti-infectives,
Leibniz Institute for Natural Product Research and Infection Biology – Hans-Knöll-Institute (HKI),
Beutenbergstraße 11a, 07745 Jena, Germany

[d] M. Müll, Dr. H. Kries
Department of Technical Biochemistry,
University of Stuttgart,
Allmandring 31, 70569 Stuttgart, Germany
*Hajo.Kries@ibc.uni-stuttgart.de

Contents

Supporting Tables 3

Supporting Figures 12

Sequences of Proteins..28

Supporting References 30

Supporting Tables

Table S1. Oligonucleotide mix for the FuncLib libraries.

Gene	Position	Oligo	Molar ratio	Residues	Oligo mix
srfAC	A660	SrfAC_660_BTT_f	3	FLV	SrfAC_660_f
		SrfAC_660_GSC_f	2	AG	
	F702	SrfAC_702_ASC_f	2	ST	SrfAC_702_f
		SrfAC_702_YAT_f	2	HY	
		SrfAC_702_TTT_f	1	F	
		SrfAC_702_TGG_f	1	W	
	C752	SrfAC_752_TGC_f	1	C	SrfAC_752_f
		SrfAC_752_DYG_f	6	ALMSTV	
grsA	A236	GrsA_236_GSA_f	2	AG	GrsA_236_f
		GrsA_236_Leu_f	1	L	
	T278	GrsA_278_BWC_f	1	FYHLDV	GrsA_278_f
		GrsA_278_DYA_f	1	LSITVA	
	A322	GrsA_322_AYG_f	1	MT	GrsA_322_f
		GrsA_322_KSC_f	2	SCAG	
		GrsA_322_SAA_f	1	QE	
		GrsA_322_VWC_f	3	LHINVD	

Table S2. PCR amplification and the assembly of fragments for FuncLib libraries.

Gene	Fragment amplification		Fragment assembly
	**Oligo (mix)**	**Fragment**	**Oligos**
srfAC	SrfAC_660_f	A	SrfAC_o_f / SrfAC_o_r
	SrfAC_660_r		
	SrfAC_702_f	B	
	SrfAC_702_r		
	SrfAC_752_f	C	
	SrfAC_o_r		
grsA	GrsA_f	A	GrsA_f / GrsA_r
	GrsA_236_r		
	GrsA_236_f	B	
	GrsA_278_r		
	GrsA_278_f	C	
	GrsA_322_r		
	GrsA_322_f	D	
	GrsA_r		

Table S3. PCR amplification and the assembly of fragments for NNK libraries of VSA.

Library	Fragment	Oligo		Restriction enzyme
**VSA-S654NNK**	654A	VSA_Blp_f	Assembly PCR	BlpI + DraIII
		VSA_S654NNK_o_r		
	654B	VSA_S654NNK_N655s_f		
		SrfAC_o_r		
**VSA-F658NNK**		VSA_F658NNK_D659s_f		BstBI + DraIII
		SrfAC_o_r		
**VSA-V660NNK**		VSA_V660NNK_F661s_f		BstBI + DraIII
		SrfAC_o_r		
**VSA-F661NNK**		VSA_F661NNK_T662s_f		BstBI + DraIII
		SrfAC_o_r		
**VSA-F663NNK**		VSA_F663NNK_D664s_f		BstBI + DraIII
		SrfAC_o_r		
**VSA-D664NNK**		VSA_D664NNK_F665s_f		BstBI + DraIII
		SrfAC_o_r		
**VSA-S702NNK**	702A	VSA_Blp_f	Assembly PCR	BlpI + DraIII
		VSA_S702NNK_o_r		
	702B	VSA_S702NNK_A703s_f		
		SrfAC_o_r		
**VSA-A703NNK**	703A	VSA_Blp_f	Assembly PCR	BlpI + DraIII
		VSA_A703NNK_o_r		
	703B	VSA_A703NNK_T704s_f		
		SrfAC_o_r		
**VSA-L726NNK**	726A	VSA_Blp_f	Assembly PCR	BlpI + DraIII
		VSA_L726NNK_o_r		
	726B	VSA_L726NNK_F727s_f		
		SrfAC_o_r		
**VSA-F727NNK**	727A	VSA_Blp_f	Assembly PCR	BlpI + DraIII
		VSA_F727NNK_o_r		
	727B	VSA_F727NNK_G728s_f		
		SrfAC_o_r		

**VSA-G728NNK**	728A	VSA_Blp_f<br>VSA_G728NNK_o_r	Assembly PCR	BlpI + DraIII
	728B	VSA_G728NNK_G729s_f<br>SrfAC_o_r		
**VSA-A752NNK**		VSA_Blp_f<br>VSA_A752NNK_N751s_r		BlpI + DraIII
**VSA-T759NNK**		VSA_Blp_f<br>VSA_T759NNK_G758s_r		BlpI + DraIII
**VSA-V760NNK**		VSA_Blp_f<br>VSA_V760NNK_T759s_r		BlpI + DraIII
**VSA-F761NNK**		VSA_Blp_f<br>VSA_F761NNK_V760s_r		BlpI + DraIII

Table S4. Oligonucleotide sequences for PCR primers. Targeted positions are labelled in bold (continued on next page).

Name	Sequence
SrfAC_o_f	GATCAGGATACGTTCTTGTCTGTTTC
SrfAC_o_r	GAATCCGGCAGATCATGCAC
SrfAC_660_BTT_f	GATCAGGATACGTTCTTGTCTGTTTCGAATTACGCCTTTGAT**BTT**TTTACCTTTGATTTCTATGC
SrfAC_660_GSC_f	GATCAGGATACGTTCTTGTCTGTTTCGAATTACGCCTTTGAT**GSC**TTTACCTTTGATTTCTATGC
SrfAC_660_r	CATGACATTGACATTCTCTTGCAG
SrfAC_702_ASC_f	CAAGAGAATGTCAATGTCATG**ASC**GCGACAACCGCACTATTTAATC
SrfAC_702_YAT_f	CAAGAGAATGTCAATGTCATG**YAT**GCGACAACCGCACTATTTAATC
SrfAC_702_TTT_f	CAAGAGAATGTCAATGTCATG**TTT**GCGACAACCGCACTATTTAATC
SrfAC_702_TGG_f	CAAGAGAATGTCAATGTCATG**TGG**GCGACAACCGCACTATTTAATC
SrfAC_702_r	GTTAATCAGCTTGCCCGGC
SrfAC_752_TGC_f	GCTGCGGATCATGGGGCCGGGCAAGCTGATTAAC**TGC**TACGGGCCGACTGAGGGAAC
SrfAC_752_DYG_f	GCTGCGGATCATGGGGCCGGGCAAGCTGATTAAC**DYG**TACGGGCCGACTGAGGGAAC
VSA_Blp_f	GATGAAAGAACAAGCGGCTGAGCTG
VSA_S654NNK_o_r	ACAGACAAGAACGTATCCTGATCAGAAAATGC
VSA_S654NNK_N655s_f	GATACGTTCTTGTCTGTT**NNKAAC**TACGCCTTTGATGTTTTTACCTTTGATTTC
VSA_F658NNK_D659s_f	GATCAGGATACGTTCTTGTCTGTTTCGAATTACGCC**NNKGACGTT**TTTACCTTTGATTTCTATGCTTCTATGC
VSA_V660NNK_F661s_f	GATCAGGATACGTTCTTGTCTGTTTCGAATTACGCCTTTGAT**NNKTTC**ACCTTTGATTTCTATGCTTCTATGCTG
VSA_F661NNK_T662s_f	GATCAGGATACGTTCTTGTCTGTTTCGAATTACGCCTTTGAT**GTTNNKACG**TTTGATTTCTATGCTTCTATGCTGAATGCG
VSA_F663NNK_D664s_f	GATCAGGATACGTTCTTGTCTGTTTCGAATTACGCCTTTGATGTTTTTACC**NNKGAC**TTCTATGCTTCTATGCTGAATGCG
VSA_D664NNK_F665s_f	GATCAGGATACGTTCTTGTCTGTTTCGAATTACGCCTTTGATGTTTTTACCTTT**NNKTTT**TATGCTTCTATGCTGAATGCGG
VSA_S702NNK_o_r	CATGACATTGACATTCTCTTGCAGG
VSA_S702NNK_A703s_f	CCTGCAAGAGAATGTCAATGTCATG**NNKGCC**ACAACCGCACTATTTAATCTTCTCAC
VSA_A703NNK_o_r	**GCT**CATGACATTGACATTCTCTTGCAGG
VSA_A703NNK_T704s_f	CCTGCAAGAGAATGTCAATGTCATG**AGCNNKACC**ACCGCACTATTTAATCTTCTCACAG
VSA_L726NNK_o_r	TATACAGCGAAGCCCCTTCATC
VSA_L726NNK_F727s_f	GATGAAGGGGCTTCGCTGTATA**NNKTTT**GGCGGAGAGCGCGCGTCAG
VSA_F727NNK_o_r	TAATATACAGCGAAGCCCCTTCATC
VSA_F727NNK_G728s_f	GATGAAGGGGCTTCGCTGTATATTA**NNKGGT**GGAGAGCGCGCGTCAGTG
VSA_G728NNK_o_r	GAATAATATACAGCGAAGCCCCTTC
pSU18_bb_f	AGATCTCATCACCATCACC
pSU18_bb_r	GGTTAATTTCTCTTTAATGAATTC
GrsA_f	AAGAGGAGAAATTAACCATGTTAA
GrsA_r	GATGGTGATGAGATCTGGA

GrsA_236_r	ATCAAAAGAGATGCTGGCAAATTGACC
GrsA_236_GSA_f	CAATTTGCCAGCATCTCTTTTGAT**GSA**TCCGTATGGGAGATGTTTATGGC
GrsA_236_Leu_f	CAATTGCCAGCATCTCTTTTGAT**TTA**TCCGTATGGGAGATGTTTATGGC
GrsA_278_r	CAGTGATTTCCTTTTGGTTAATGTATTG
GrsA_278_BWC_f	CATTAACCAAAAGGAAATCACTGTTATT**BWC**TTGCCACCTACCTATGTAG
GrsA_278_DYA_f	CATTAACCAAAAGGAAATCACTGTTATT**DYA**TTGCCACCTACCTATGTAG
GrsA_322_r	ATTTATGTAAGTTACTTTCTCCTTCCA
GrsA_322_AYG_f	GGAAGGAGAAAGTAACTTACATAAAT**AYG**TACGGCCCTACGGAAACAAC
GrsA_322_KSC_f	GGAAGGAGAAAGTAACTTACATAAAT**KSC**TACGGCCCTACGGAAACAAC
GrsA_322_SAA_f	GGAAGGAGAAAGTAACTTACATAAAT**SAA**TACGGCCCTACGGAAACAAC
GrsA_322_VWC_f	GGAAGGAGAAAGTAACTTACATAAAT**VWC**TACGGCCCTACGGAAACAAC

Table S5. Mutant coverage in NNK libraries of VSA. Numbers in the table denote the frequency of occurrence of the mutant. Missing mutants that were cloned individually with non-degenerate primers are marked in red.

	Mutation																				
**Position**	**A**	**R**	**N**	**D**	**C**	**Q**	**E**	**G**	**H**	**I**	**L**	**K**	**M**	**F**	**P**	**S**	**T**	**W**	**Y**	**V**	**Missing**
**S654**	3	7	0	2	5	0	3	5	0	4	13	0	2	9	0	10	0	6	3	8	NQHKPT
**F658**	1	5	3	3	6	3	2	5	0	4	7	3	1	8	2	9	2	5	1	9	H
**V660**	1	5	1	3	7	1	1	9	4	6	13	0	1	4	1	4	2	3	4	8	K
**F661**	3	3	0	2	0	2	4	10	4	0	5	4	3	7	7	5	1	4	5	11	NCI
**F663**	1	6	5	2	0	7	2	6	0	1	10	4	3	4	5	1	2	5	0	2	CHY
**D664**	0	12	5	6	0	4	1	18	0	1	4	0	3	2	4	2	3	0	0	12	ACHKWY
**S702**	0	4	1	3	1	0	3	8	0	4	8	1	5	10	1	12	0	4	3	7	AQHT
**A703**	5	5	0	2	2	0	3	7	4	3	7	1	1	5	1	3	1	7	5	13	NQ
**L726**	2	4	6	1	5	0	4	4	2	7	7	4	2	8	0	5	2	5	3	6	QP
**F727**	2	1	3	3	6	0	0	5	2	2	14	1	5	11	0	2	3	5	4	7	QEP
**G728**	2	2	3	2	6	2	2	5	0	4	11	0	5	12	0	3	0	3	3	12	HKPT
**A752**	10	6	1	2	4	1	3	1	3	0	11	6	1	3	11	4	4	0	0	1	IWY
**T759**	1	7	9	0	1	4	1	0	4	5	5	8	0	5	8	8	8	1	5	3	DGM
**V760**	2	4	2	4	0	2	0	0	4	1	10	10	2	2	6	6	6	1	3	5	CEG
**F761**	4	2	2	7	1	2	0	2	3	4	7	3	6	5	9	7	4	2	5	5	E

Table S6. Top 20 mutants from FuncLib SrfAC library and VSA NNK libraries with highest activity (A_{rel}), promiscuity and selectivity (I_{rel}) relative to the progenitor SrfAC and VSA, respectively.

SrfAC FuncLib library				VSA NNK libraries					
Activity		Promiscuity		Activity		Promiscuity		Specificity	
Mutant	A_{rel}	Mutant	I_{rel}	Mutant	A_{rel}	Mutant	I_{rel}	Mutant	I_{rel}
ASV	3.35	VYS	2.77	A752I	2.27	S702F#	1.46	G728M	0.06
ASA	2.96	GWV	2.72	S702T	2.21	V660E	1.33	G728L	0.09
VSA	2.27	ASA	2.66	V660L	2.08	S654I	1.29	V660W	0.10
VSV	2.03	GWS	2.62	S702A	2.04	V660Q	1.28	A752M	0.13
ASL	1.91	ASV	2.45	V660I	1.55	F658Q	1.27	V660Y	0.14
VSL	1.60	ASL	2.34	A703N	1.54	V660S	1.27	V660F	0.21
LSL	1.51	AWS	2.31	V660A	1.51	V660A	1.27	F761A	0.27
GSL	1.46	AWM	2.28	A703I	1.51	F658A	1.26	G728A	0.29
VFA	1.39	VSA	2.24	F663W	1.51	S654Q	1.26	F727Y	0.41
GTV	1.28	VFA	2.19	A703M	1.42	F658S	1.25	S702D	0.41
GSV	1.22	GWC	2.14	F661A	1.41	F663F	1.24	L726D	0.48
GTL	1.15	FWL	2.00	S654N	1.37	F658G	1.24	L726G	0.50
GSC	1.07	FFA	1.98	V760G	1.33	A752G	1.22	F727S	0.50
GST	1.05	VSV	1.91	A703L	1.24	F658T	1.21	G728F	0.51
VFM	1.03	LWA	1.86	A752V	1.24	S654L	1.21	L726A	0.57
AFC	1.02	GYM	1.69	S654A	1.23	D664E	1.20	F761I	0.57
FSV	0.99	FWS	1.66	S654G	1.19	S654G	1.20	F727A	0.57
GYV	0.98	AWL	1.63	F727I	1.15	F661T	1.20	F761V	0.58
FSA	0.96	VYL	1.63	A703A	1.10	S654M	1.20	L726Y	0.58
FSL	0.96	GSL	1.54	V660G	1.07	F658H	1.19	F727T	0.58

#The high promiscuity of this mutant could not be confirmed with protein purified on large scale.

Table S7. Specificity codes of SrfAC and mutants compared to natural specificity codes. According to the measured specificity, mutants have been classified into A-domain types. For natural A-domains of the same type according to a database of specificities and codes [1], the frequency of occurrence of residues at the specified positions is noted as percentage. Residue identities that are absent (0 %), rare (0 - 10 %), or frequent (>10 %) are marked in red, yellow, and light green, respectively.

		Specificity code position in SrfAC numbering<br>Second row: percentage in natural codes									
**Enzyme**	**Measured specificity**	**659**	**660**	**663**	**702**	**726**	**728**	**752**	**760**	**761**	**A-domain type according to Rausch et al.** [1]
**GrsA**	Phe	D	A	W	T	I	A	A	I	C	
**SrfAC**	Leu	D	A	F	F	L	G	C	V	F	
**VSA**	multi	D	V<br>2%	F	S<br>22%	L	G	A<br>83%	V	F	nonpolar
**Met-specific**	Met	D	M/I/L	F	S	L	G	A	V	F	-
**A752G**	Phe	D	V<br>0%	F	S<br>0%	L	G	G<br>23%	V	F	Phe
**V660A**	aromatic	D	A	F	S<br>7%	L	G	A<br>64%	V	F	aromatic
**S702A**	aromatic	D	V<br>1.5%	F	A<br>6%	L	G	A<br>64%	V	F	aromatic
**V760G**	D/L-Phe	D	V<br>0%	F	S<br>0%	L	G	A<br>77%	G<br>0%	F	Phe
**G728A**	Ala	D	V<br>3%	F	S<br>0%	L	A<br>37%	A<br>10%	V	F	small
**G728M**	Ala	D	V<br>3%	F	S<br>0%	L	M<br>0%	A<br>10%	V	F	small
**V660W**	Leu	D	W<br>0%	F	S<br>0%	L	G	A<br>10%	V	F	Leu
**V660F**	Leu	D	F<br>1%	F	S<br>0%	L	G	A<br>10%	V	F	Leu

Supporting Figures

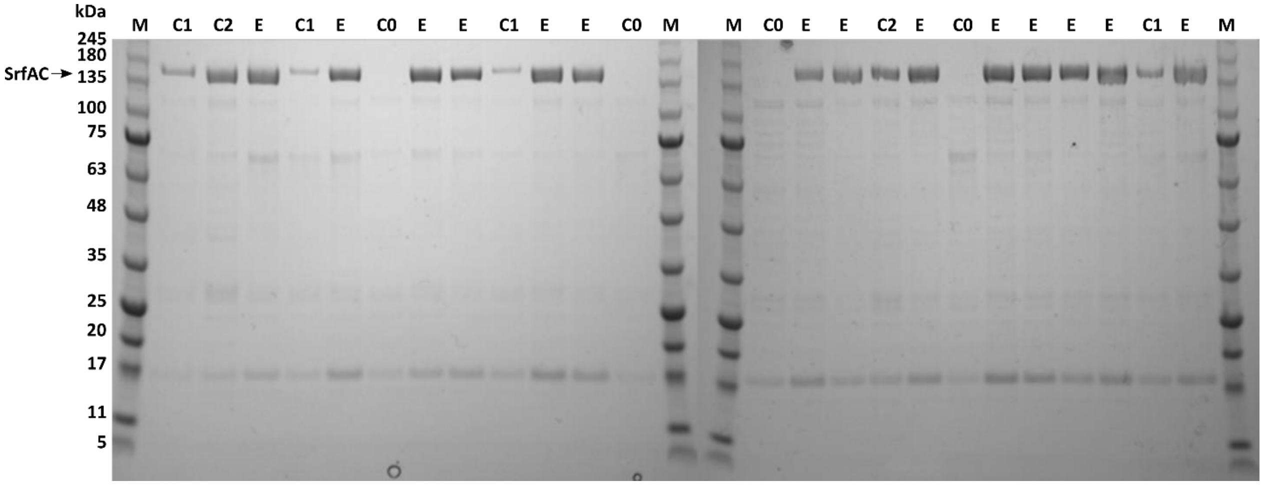

Figure S1. SDS PAGE of SrfAC expressed and purified in 96-well plate format. Expected size: 143.9 kDa. Proteins were eluted from magnetic beads with 200 µL of elution buffer (50 mM Tris pH 8.0, 200 mM imidazole) and 5 µL was loaded on the gel. E, HM0079 strain with pTrc99a-SrfAC; C0, negative control containing the empty vector; C1, purification control with empty vector and SrfAC added to the cell lysate; C2, purification control with empty vector and SrfAC added to the eluate; M, Triple Color Protein Standard III (Serva). Bolt 4-12% Bis-Tris Plus Gels (ThermoFisher Scientific) with MES-SDS running buffer (Novex) were run at 200 V for 22 min and stained with Quick Coomassie stain (Serva).

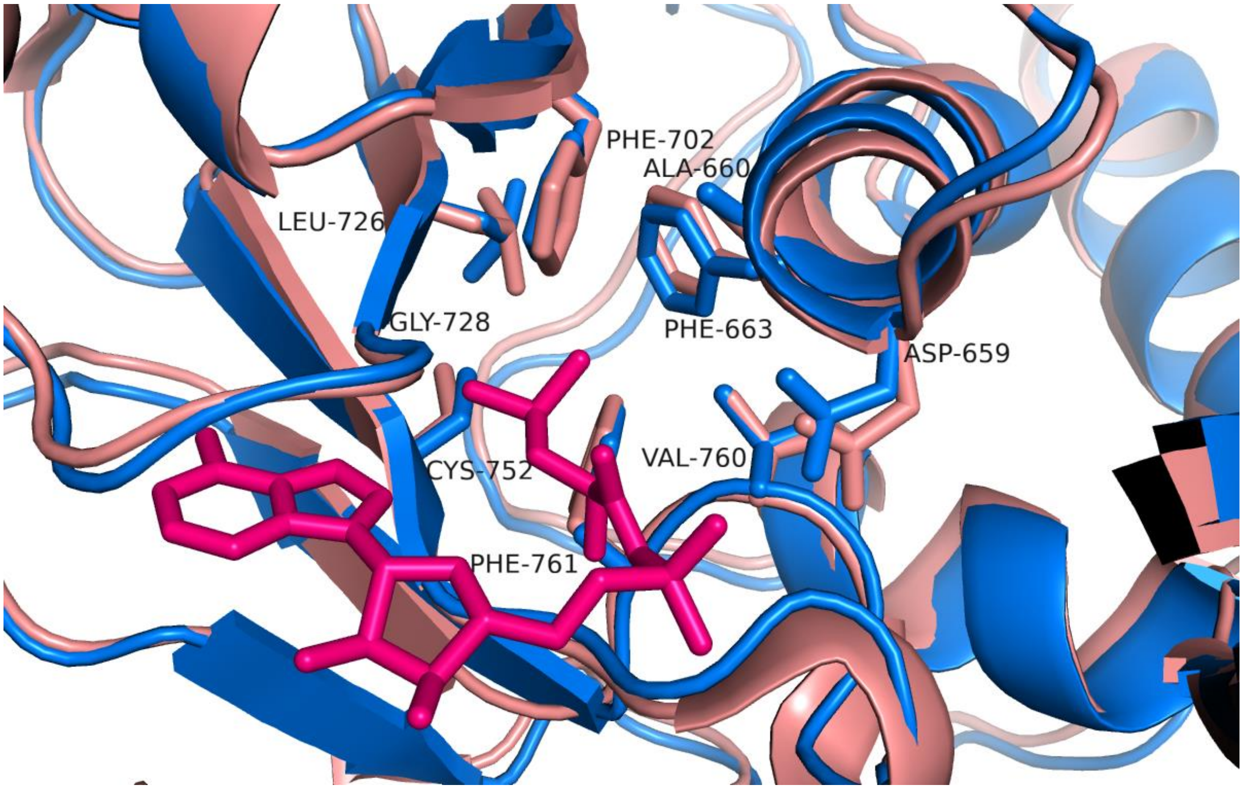

Figure S2. Overlay of YASARA model of SrfAC with Leu-AMP (blue) and SrfAC crystal structure (PDB: 2VSQ, pink). Specificity code residues are labeled.

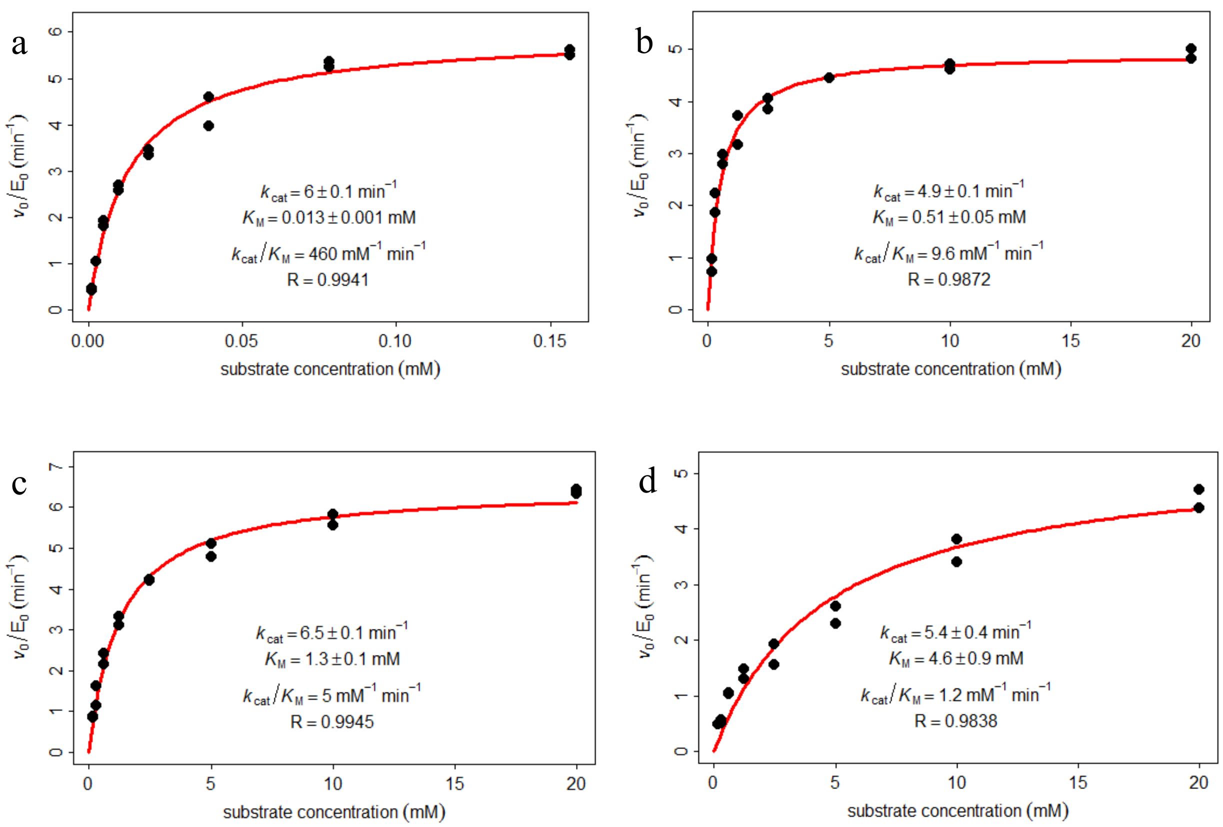

Figure S3. Saturation kinetics of SrfAC with L-Leu (a) and VSA with L-Leu (b), L-Phe (c) and L-Met (d) measured with the MesG/hydroxylamine spectrophotometric assay. Assays were conducted with a single enzyme batch in technical duplicates.

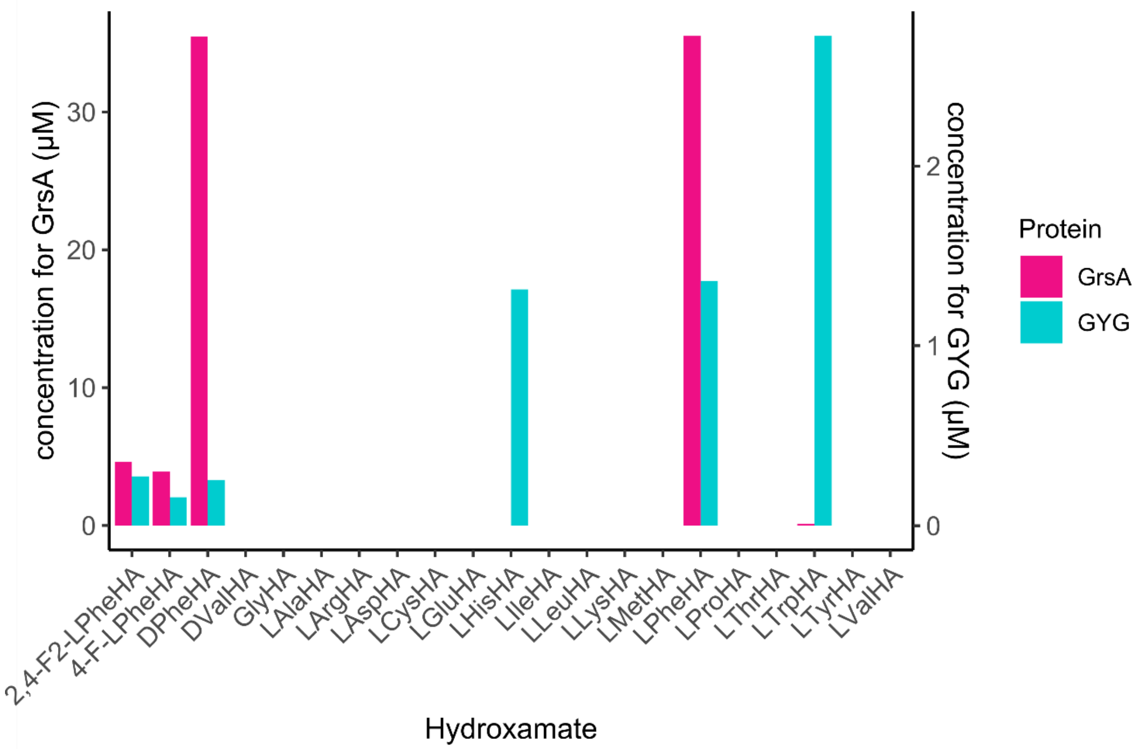

Figure S4. Enzymatic activity of GrsA and variant GYG measured using the hydroxamate multiplex assay (HAMA). GrsA enzyme variants were engineered through directed evolution and screened for altered substrate specificity giving rise to GYG. Hydroxamate formation has been analyzed using LC-MS/MS and hydroxamate concentrations are plotted for GrsA (pink) and G. The concentration of hydroxamate products for GrsA and GYG is compared with distinct y-axis scales to account for differences in activity levels. Additionally, the assay included two fluorinated Phe derivatives [2] to assess enzyme specificity towards these modified substrates.

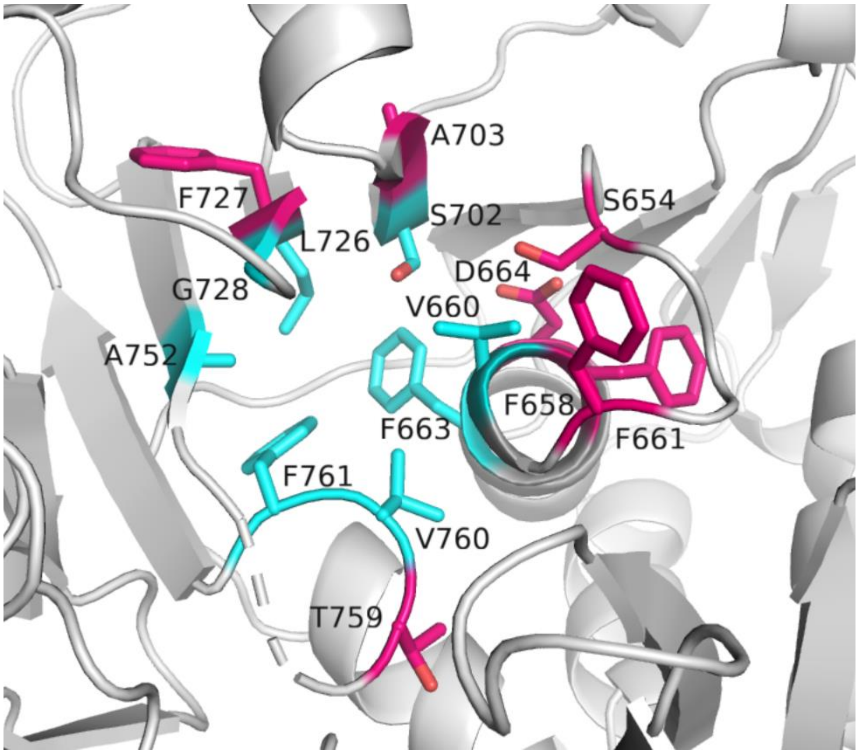

Figure S5. Residues in the binding pocket of VSA model selected for saturation mutagenesis. The specificity code residues in the first shell are shown in cyan and in the second shell in pink. The VSA structure is a SWISS homology model built against SrfAC (PDB: 2vsq) as a template.

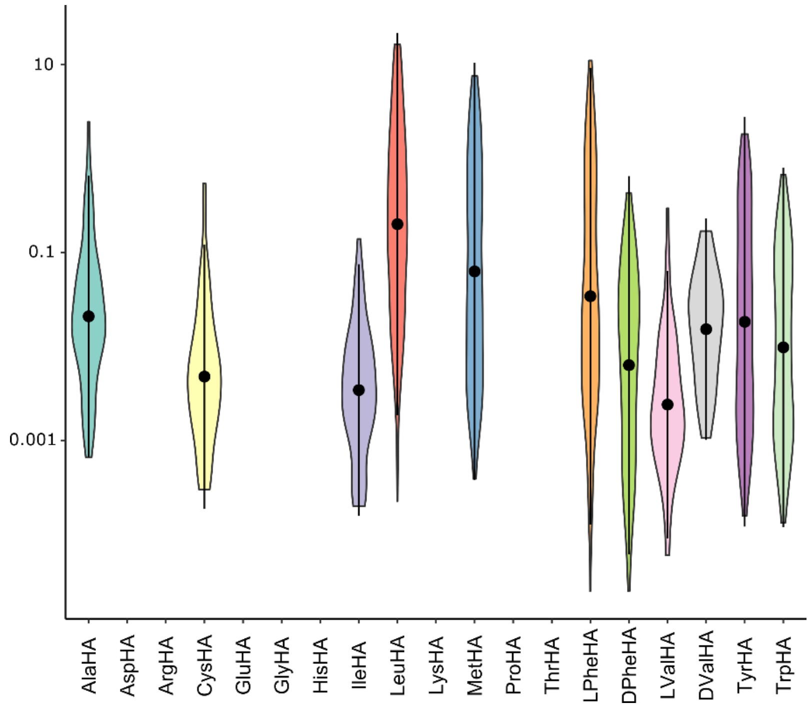

Figure S6. Logarithmic distribution of concentration (μM) of detected hydroxamates pooled from 15 NNK libraries.

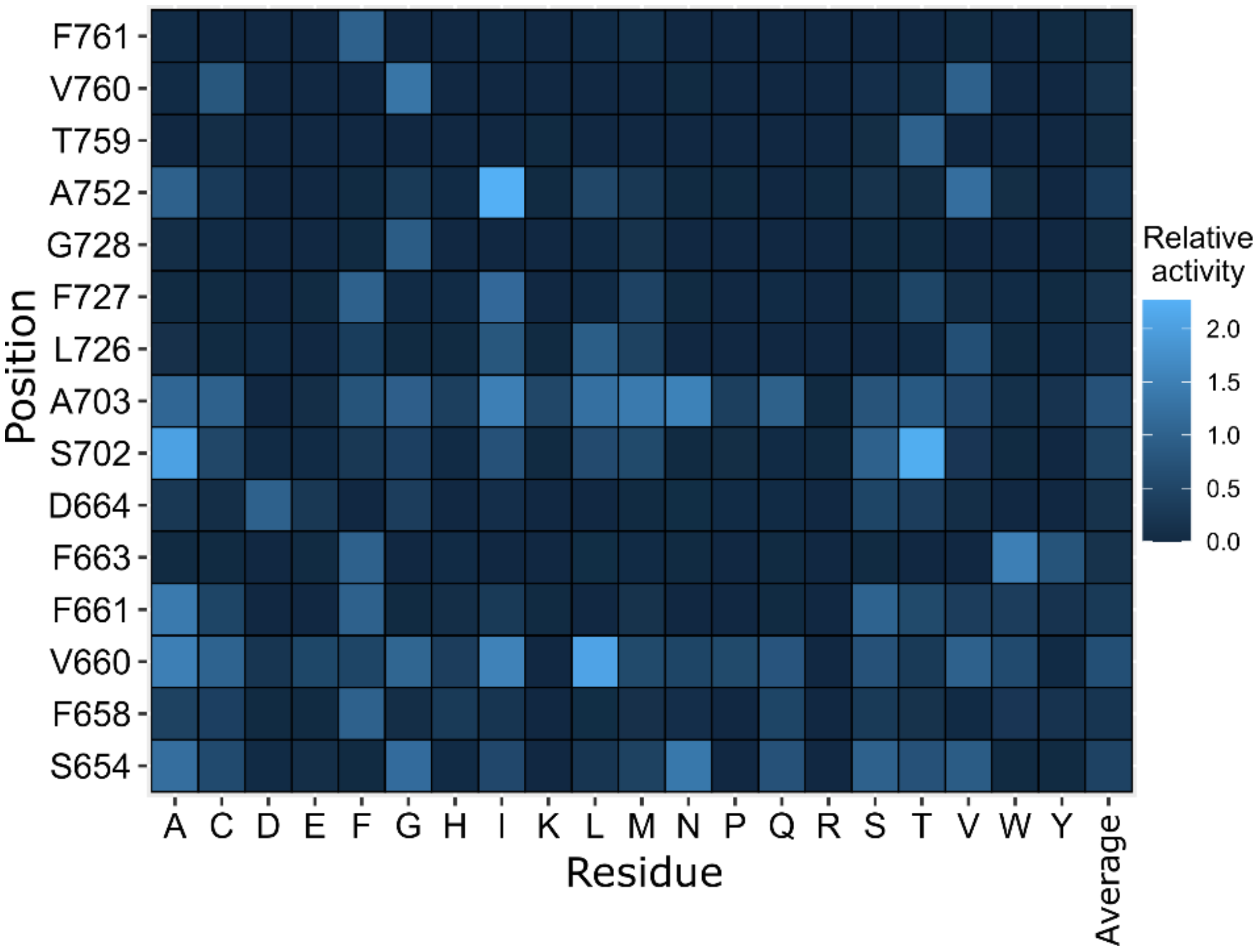

Figure S7. Heatmap of activities of all mutants from 15 NNK libraries relative to the progenitor VSA. Activity is calculated as a sum of all formed hydroxamates per mutant. Last column represents the average activity per position.

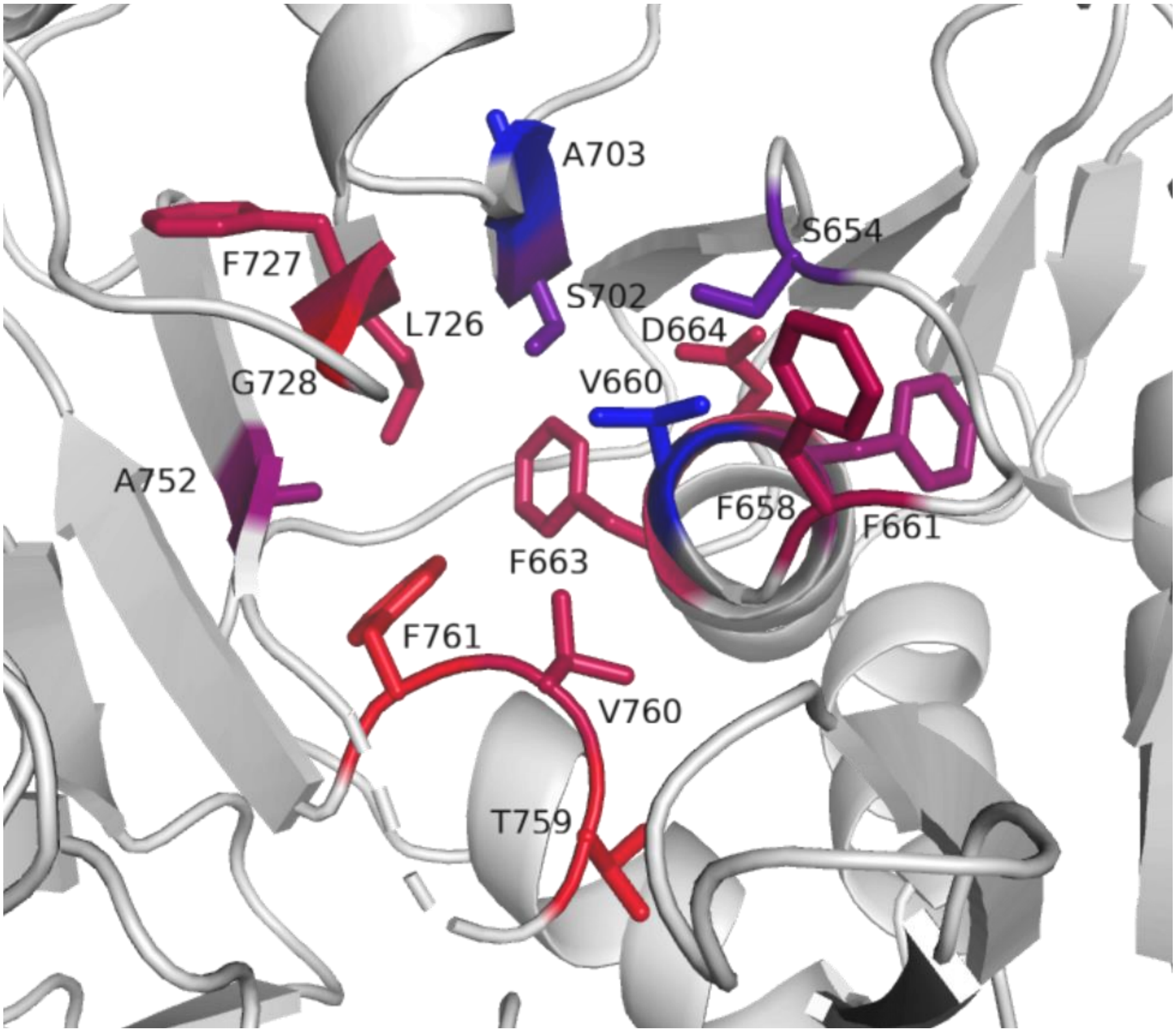

Figure S8. Binding pocket of VSA homology model with targeted residues colored according to the average activity per position, relative to the progenitor VSA. Mutations at blue positions result in highest activities and at red positions, lowest.

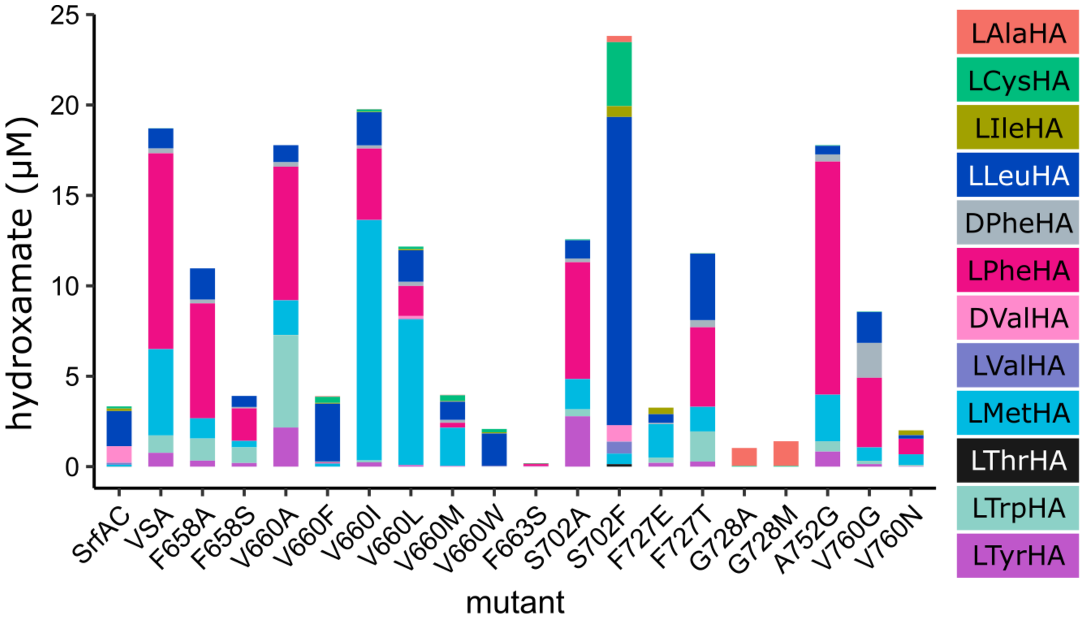

Figure S9. HAMA profiles of SrfAC, VSA, and mutants. Fractions of hydroxamates are means from three technical replicates from two batches of enzyme. Enzyme reactions were incubated for 60 min at 25 ºC and 1 µM enzyme. The plot is a different representation of the data shown in Figure 4.

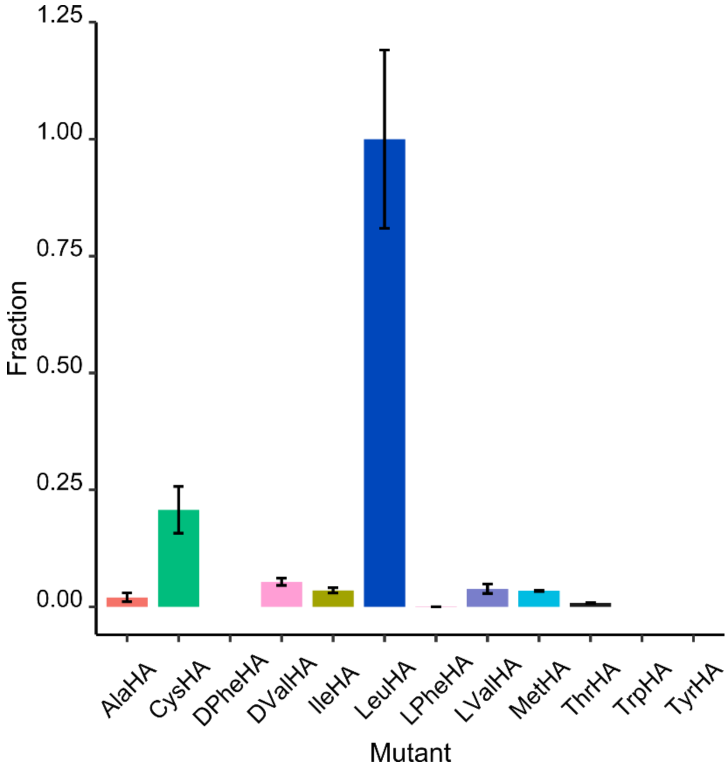

Figure S10. HAMA profile of S702F mutant of VSA. Error bars are standard deviations from three technical replicates from two batches of enzyme. Enzyme reactions were incubated for 60 min at 25 ºC and 1 μM enzyme.

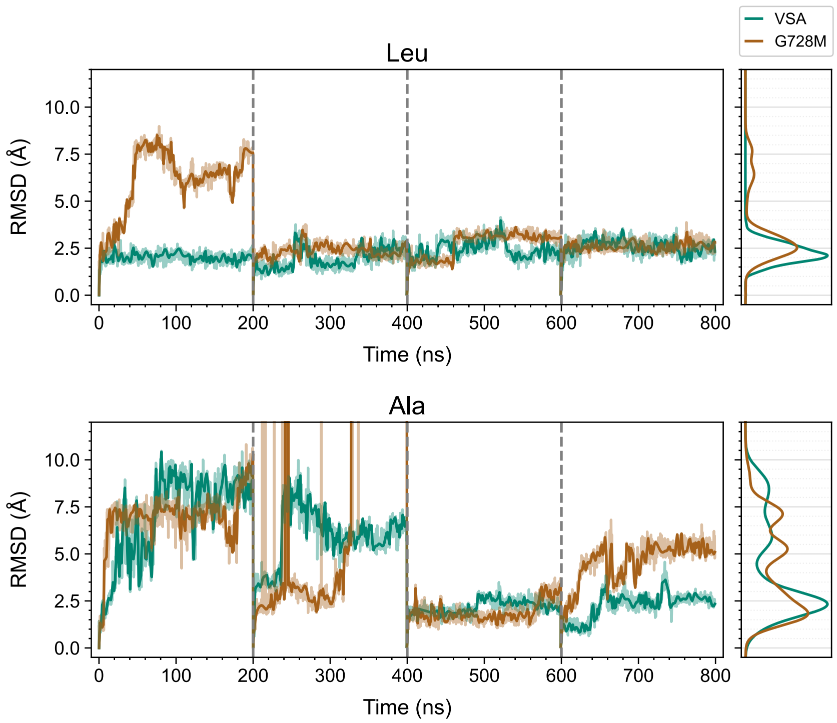

Figure S11. Variation of substrate RMSD along MD simulations. Four replicates of 200 ns were run for each system. Righthand side plots show the respective density distributions.

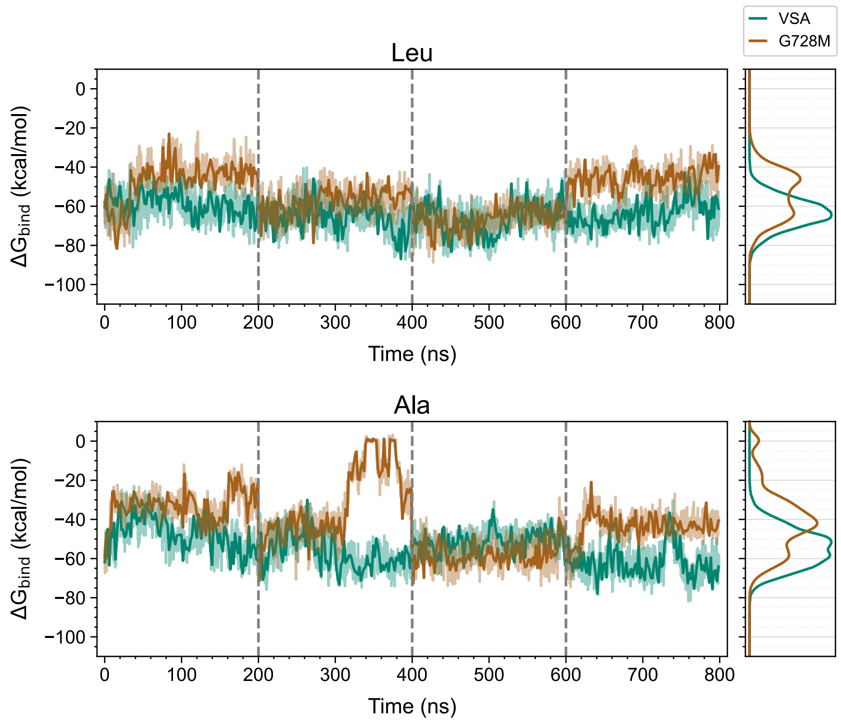

Figure S12. Binding free energy calculated by MM/GBSA method for each MD frame. Four replicates of 200 ns were run for each system. Righthand side plots show the respective density distributions.

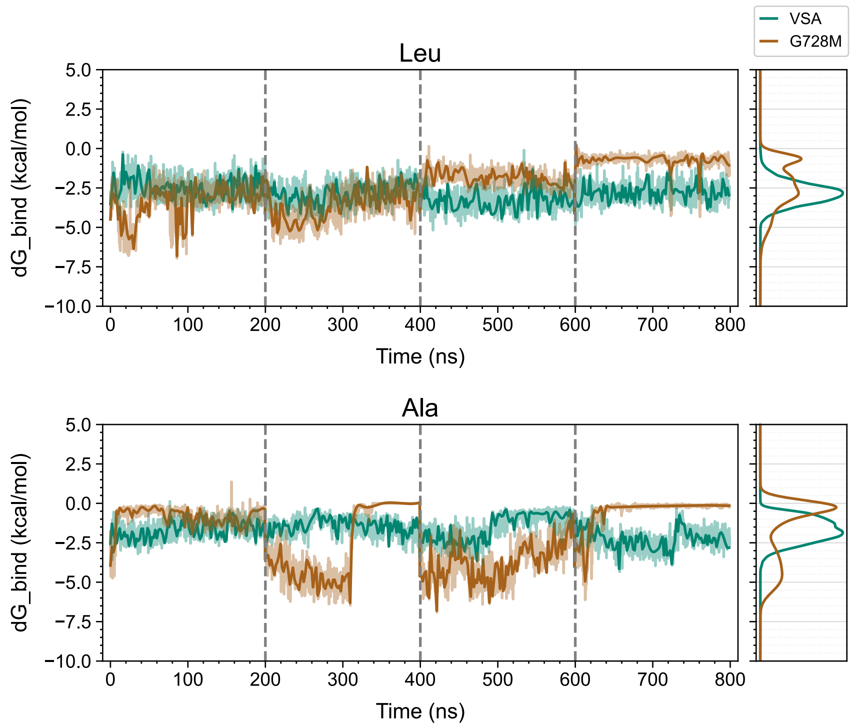

Figures S13. Energy contributions of residue 728 to the binding free energy calculated by MM/GBSA method for each MD frame. Four replicates of 200 ns were run for each system. Righthand side plots show the respective density distributions.

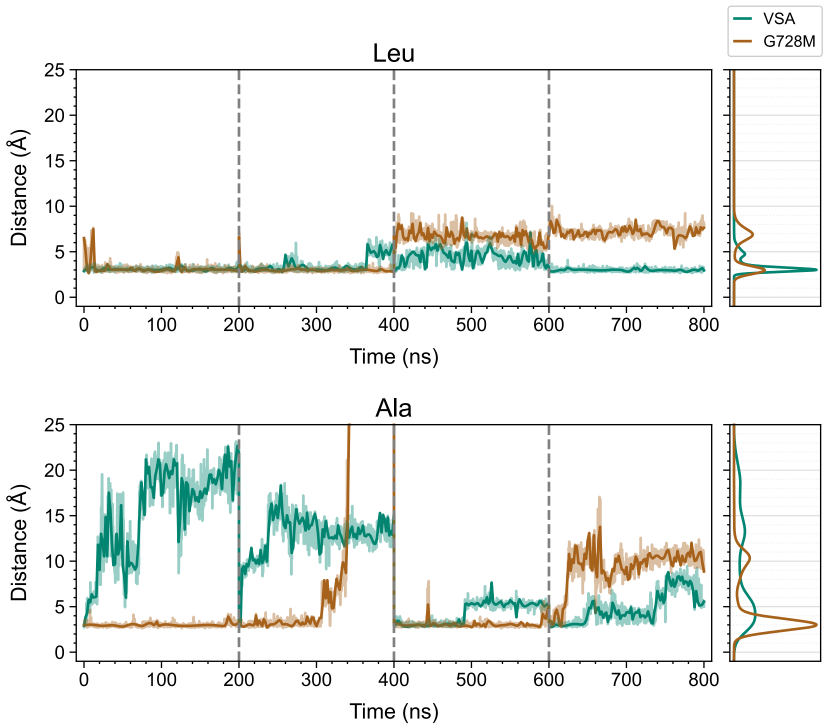

Figure S14. Distance between C=O group of V760 and substrate N (in NH_3^+). Four replicates of 200 ns were run for each system. Righthand side plots show the respective density distributions.

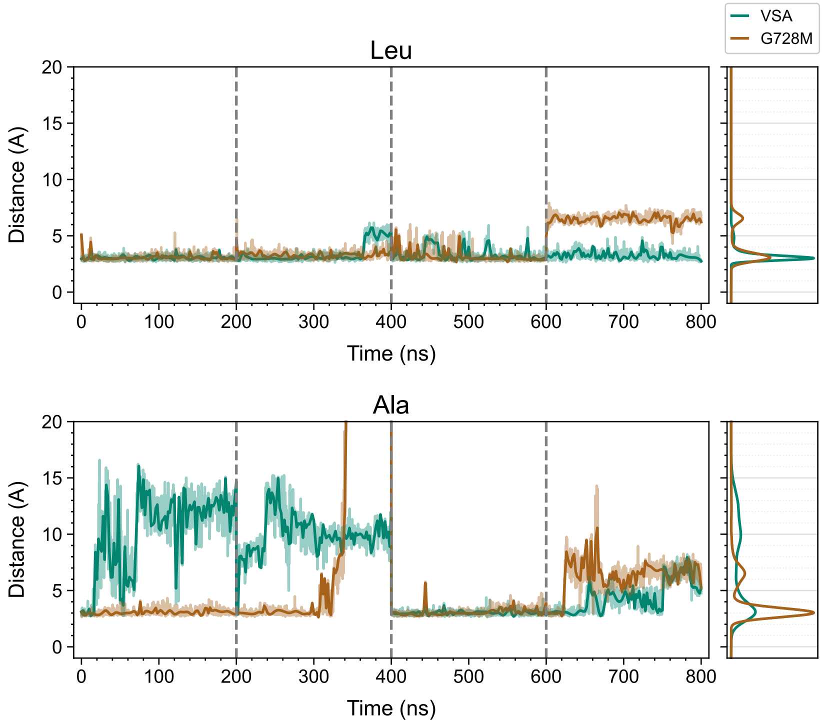

Figure S15. Distance between the C=O group of G754 and substrate N (in NH_3^+). Four replicates of 200 ns were run for each system. Righthand side plots show the respective density distributions.

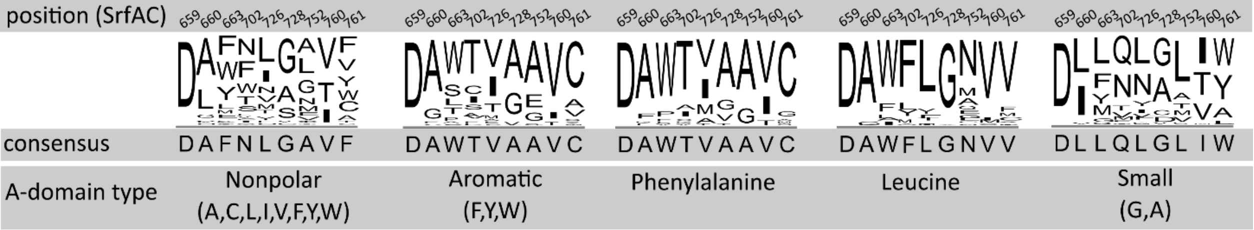

Figure S16. Consensus sequences of specificity codes of A-domains activating different amino acid substrates. Alignments are generated with Muscle [3] using a curated specificity code database from Rausch et al. [1].

Sequences of Proteins

The A-domain is highlighted in blue. The residues mutated in the promiscuous (VSA or GYG) mutant are highlighted in bold, randomized residues in the specificity code in red, and those in the second shell in yellow.

SrfAC

MSQFSKDQVQDMYYLSPMQEGMLFHAILNPGQSFYLEQITMKVKGSLNIKCLEESMNVIMDRYDVFRTVFIHEKVKRPVQVVLKKRQ
FHIEEIDLTHLTGSEQTAKINEYKEQDKIRGFDLTRDIPMRAAIFKKAEESFEWVWSYHHIILDGWCFGIVVQDLFKVYNALREQKP
YSLPPVKPYKDYIKWLEKQDKQASLRYWREYLEGFEGQTTFAEQRKKQKDGYEPKELLFSLSEAETKAFTELAKSQHTTLSTALQAV
WSVLISRYQQSGDLAFGTVVSGRPAEIKGVEHMVGLFINVVPRRVKLSEGITFNGLLKRLQEQSLQSEPHQYVPLYDIQSQADQPKL
IDHIIVFENYPLQDAKNEESSENGFDMVDVHVFEKSNYDLNLMASPGDEMLIKLAYNENVFDEAFILRLKSQLLTAIQQLIQNPDQP
VSTINLVDDREREFLLTGLNPPAQAHETKPLTYWFKEAVNANPDAPALTYSGQTLSYRELDEEANRIARRLQKHGAGKGSVVALYTK
RSLELVIGILGVLKAGAAYLPVDPKLPEDRISYMLADSAAACLLTHQEMKEQAAELPYTGTTLFIDDQTRFEEQASDPATAIDPNDP
AYIMYTSGTTGKPKGNITTHANIQGLVKHVDYMAFSDQDTFLSVSNYAFDAFTFDFYASMLNAARLIIADEHTLLDTERLTDLILQE
NVNVMFATTALFNLLTDAGEDWMKGLRCILFGGERASVPHVRKALRIMGPGKLINCYGPTEGTVFATAHVVHDLPDSISSLPIGKPI
SNASVYILNEQSQLQPFGAVGELCISGMGVSKGYVNRADLTKEKFIENPFKPGETLYRTGDLARWLPDGTIEYAGRIDDQVKIRGHR
IELEEIEKQLQEYPGVKDAVVVADRHESGDASINAYLVNRTQLSAEDVKAHLKKQLPAYMVPQTFTFLDELPLTTNGKVNKRLLPKP
DQDQLAEEWIGPRNEMEETIAQIWSEVLGRKQIGIHDDFFALGGHSLKAMTAASRIKKELGIDLPVKLLFEAPTIAGISAYLKNGGS
DGLQDVTIMNQDQEQIIFAFPPVLGYGLMYQNLSSRLPSYKLCAFDFIEEEDRLDRYADLIQKLQPEGPLTLFGYSAGCSLAFEAAK
KLEEQGRIVQRIIMVDSYKKQGVSDLDGRTVESDVEALMNVNRDNEALNSEAVKHGLKQKTHAFYSYYVNLISTGQVKADIDLLTSG
ADFDMPEWLASWEEATTGVYRVKRGFGTHAEMLQGETLDRNAEILLEFLNTQTVTVSGSRSHHHHHH

SrfAC-VSA

MSQFSKDQVQDMYYLSPMQEGMLFHAILNPGQSFYLEQITMKVKGSLNIKCLEESMNVIMDRYDVFRTVFIHEKVKRPVQVVLKKRQ
FHIEEIDLTHLTGSEQTAKINEYKEQDKIRGFDLTRDIPMRAAIFKKAEESFEWVWSYHHIILDGWCFGIVVQDLFKVYNALREQKP
YSLPPVKPYKDYIKWLEKQDKQASLRYWREYLEGFEGQTTFAEQRKKQKDGYEPKELLFSLSEAETKAFTELAKSQHTTLSTALQAV
WSVLISRYQQSGDLAFGTVVSGRPAEIKGVEHMVGLFINVVPRRVKLSEGITFNGLLKRLQEQSLQSEPHQYVPLYDIQSQADQPKL
IDHIIVFENYPLQDAKNEESSENGFDMVDVHVFEKSNYDLNLMASPGDEMLIKLAYNENVFDEAFILRLKSQLLTAIQQLIQNPDQP
VSTINLVDDREREFLLTGLNPPAQAHETKPLTYWFKEAVNANPDAPALTYSGQTLSYRELDEEANRIARRLQKHGAGKGSVVALYTK
RSLELVIGILGVLKAGAAYLPVDPKLPEDRISYMLADSAAACLLTHQEMKEQAAELPYTGTTLFIDDQTRFEEQASDPATAIDPNDP
AYIMYTSGTTGKPKGNITTHANIQGLVKHVDYMAFSDQDTFLSVSNYAFD**V**FTFDFYASMLNAARLIIADEHTLLDTERLTDLILQE
NVNVM**S**ATTALFNLLTDAGEDWMKGLRCILFGGERASVPHVRKALRIMGPGKLIN**A**YGPTEGTVFATAHVVHDLPDSISSLPIGKPI
SNASVYILNEQSQLQPFGAVGELCISGMGVSKGYVNRADLTKEKFIENPFKPGETLYRTGDLARWLPDGTIEYAGRIDDQVKIRGHR
IELEEIEKQLQEYPGVKDAVVVADRHESGDASINAYLVNRTQLSAEDVKAHLKKQLPAYMVPQTFTFLDELPLTTNGKVNKRLLPKP
DQDQLAEEWIGPRNEMEETIAQIWSEVLGRKQIGIHDDFFALGGHSLKAMTAASRIKKELGIDLPVKLLFEAPTIAGISAYLKNGGS
DGLQDVTIMNQDQEQIIFAFPPVLGYGLMYQNLSSRLPSYKLCAFDFIEEEDRLDRYADLIQKLQPEGPLTLFGYSAGCSLAFEAAK
KLEEQGRIVQRIIMVDSYKKQGVSDLDGRTVESDVEALMNVNRDNEALNSEAVKHGLKQKTHAFYSYYVNLISTGQVKADIDLLTSG
ADFDMPEWLASWEEATTGVYRVKRGFGTHAEMLQGETLDRNAEILLEFLNTQTVTVSGSRSHHHHHH

GrsA

MLNSSKSILIHAQNKNGTHEEEQYLFAVNNTKAEYPRDKTIHQLFEEQVSKRPNNVAIVCENEQLTYHELNVKANQLARIFIEKGIG
KDTLVGIMMEKSIDLFIGILAVLKAGGAYVPIDIEYPKERIQYILDDSQARMLLTQKHLVHLIHNIQFNGQVEIFEEDTIKIREGTN
LHVPSKSTDLAYVIYTSGTTGNPKGTMLEHKGISNLKVFFENSLNVTEKDRIGQFASISFDASVWEMFMALLTGASLYIILKDTIND
FVKFEQYINQKEITVITLPPTYVVHLDPERILSIQTLITAGSATSPSLVNKWKEKVTYINAYGPTETTICATTWVATKETIGHSVPI
GAPIQNTQIYIVDENLQLKSVGEAGELCIGGEGLARGYWKRPELTSQKFVDNPFVPGEKLYKTGDQARWLSDGNIEYLGRIDNQVKI
RGHRVELEEVESILLKHMYISETAVSVHKDHQEQPYLCAYFVSEKHIPLEQLRQFSSEELPTYMIPSYFIQLDKMPLTSNGKIDRKQ
LPEPDLTFGMRVDYEAPRNEIEETLVTIWQDVLGIEKIGIKDNFYALGGDSIKAIQVAARLHSYQLKLETKDLLKYPTIDQLVHYIK
DSKRRSEQGIVEGEIGLTPIQHWFFEQQFTNMHHWNQSYMLYRPNGFDKEILLRVFNKIVEHHDALRMIYKHHNGKIVQINRGLEGT
LFDFYTFDLTANDNEQQVICEESARLQNSINLEVGPLVKIALFHTQNGDHLFMAIHHLVVDGISWRILFEDLATAYEQAMHQQTIAL
PEKTDSFKDWSIELEKYANSELFLEEAEYWHHLNYYTENVQIKKDYVTMNNKQKNIRYVGMELTIEETEKLLKNVNKAYRTEINDIL
LTALGFALKEWADIDKIVINLEGHGREEILEQMNIARTVGWFTSQYPVVLDMQKSDDLSYQIKLMKENLRRIPNKGIGYEIFKYLTT
EYLRPVLPFTLKPEINFNYLGQFDTDVKTELFTRSPYSMGNSLGPDGKNNLSPEGESYFVLNINGFIEEGKLHITFSYNEQQYKEDT
IQQLSRSYKQHLLAIIEHCVQKEDTELTPSDFSFKELELEEMDDIFDLLADSLTGSRSHHHHHH

GrsA-GYG

MLNSSKSILIHAQNKNGTHEEEQYLFAVNNTKAEYPRDKTIHQLFEEQVSKRPNNVAIVCENEQLTYHELNVKANQLARIFIEKGIG
KDTLVGIMMEKSIDLFIGILAVLKAGGAYVPIDIEYPKERIQYILDDSQARMLLTQKHLVHLIHNIQFNGQVEIFEEDTIKIREGTN
LHVPSKSTDLAYVIYTSGTTGNPKGTMLEHKGISNLKVFFENSLNVTEKDRIGQFASISFD**G**SVWEMFMALLTGASLYIILKDTIND
FVKFEQYINQKEITVI**Y**LPPTYVVHLDPERILSIQTLITAGSATSPSLVNKWKEKVTYIN**G**YGPTETTICATTWVATKETIGHSVPI
GAPIQNTQIYIVDENLQLKSVGEAGELCIGGEGLARGYWKRPELTSQKFVDNPFVPGEKLYKTGDQARWLSDGNIEYLGRIDNQVKI
RGHRVELEEVESILLKHMYISETAVSVHKDHQEQPYLCAYFVSEKHIPLEQLRQFSSEELPTYMIPSYFIQLDKMPLTSNGKIDRKQ
LPEPDLTFGMRVDYEAPRNEIEETLVTIWQDVLGIEKIGIKDNFYALGGDSIKAIQVAARLHSYQLKLETKDLLKYPTIDQLVHYIK
DSKRRSEQGIVEGEIGLTPIQHWFFEQQFTNMHHWNQSYMLYRPNGFDKEILLRVFNKIVEHHDALRMIYKHHNGKIVQINRGLEGT
LFDFYTFDLTANDNEQQVICEESARLQNSINLEVGPLVKIALFHTQNGDHLFMAIHHLVVDGISWRILFEDLATAYEQAMHQQTIAL
PEKTDSFKDWSIELEKYANSELFLEEAEYWHHLNYYTENVQIKKDYVTMNNKQKNIRYVGMELTIEETEKLLKNVNKAYRTEINDIL
LTALGFALKEWADIDKIVINLEGHGREEILEQMNIARTVGWFTSQYPVVLDMQKSDDLSYQIKLMKENLRRIPNKGIGYEIFKYLTT
EYLRPVLPFTLKPEINFNYLGQFDTDVKTELFTRSPYSMGNSLGPDGKNNLSPEGESYFVLNINGFIEEGKLHITFSYNEQQYKEDT
IQQLSRSYKQHLLAIIEHCVQKEDTELTPSDFSFKELELEEMDDIFDLLADSLTGSRSHHHHHH

Supporting References

(1) Rausch, C.; Weber, T.; Kohlbacher, O.; Wohlleben, W.; Huson, D. H. Specificity Prediction of Adenylation Domains in Nonribosomal Peptide Synthetases (NRPS) Using Transductive Support Vector Machines (TSVMs). *Nucleic Acids Res.* **2005**, *33* (18), 5799–5808. https://doi.org/10.1093/nar/gki885.

(2) Müll, M.; Pourmasoumi, F.; Wehrhan, L.; Nosovska, O.; Stephan, P.; Zeihe, H.; Vilotijevic, I.; Keller, B. G.; Kries, H. Biosynthetic Incorporation of Fluorinated Amino Acids into the Nonribosomal Peptide Gramicidin S. *RSC Chem. Biol.* **2023**, 10.1039.D3CB00061C. https://doi.org/10.1039/D3CB00061C.

(3) Edgar, R. C. MUSCLE: A Multiple Sequence Alignment Method with Reduced Time and Space Complexity. *BMC Bioinformatics* **2004**, *5* (1), 113. https://doi.org/10.1186/1471-2105-5-113.
